# Microfluidic Production of Spatially Structured Biomimetic Microgels as Compartmentalized Artificial Cells

**DOI:** 10.1002/smsc.202400320

**Published:** 2025-02-06

**Authors:** Matthew E. Allen, James W. Hindley, Robert V. Law, Oscar Ces, Yuval Elani

**Affiliations:** ^1^ Department of Chemistry Imperial College London Molecular Sciences Research Hub, 82 Wood Lane London W12 0BZ UK; ^2^ Institute of Chemical Biology Imperial College London Molecular Sciences Research Hub, 82 Wood Lane London W12 0BZ UK; ^3^ Department of Chemical Engineering Imperial College London Exhibition Road, South Kensington London SW7 2AZ UK; ^4^ FabriCELL Imperial College London Molecular Sciences Research Hub, 82 Wood Lane London W12 0BZ UK

**Keywords:** artificial cells, biomimicry, compartmentalization, hydrogels, microfluidics

## Abstract

Artificial cells serve as promising micro‐robotic platforms that replicate cellular features. One ubiquitous characteristic of living cells is compartmentalization of content in distinct and well‐defined locations. Herein, a microfluidic strategy to mimic compartmentalization is developed through the production of micron‐scale two and three compartment biomimetic microgels, where hydrogel compartment number, composition, size, and shape can be controlled. Our lab‐on‐chip system enables the incorporation of various synthetic organelles into spatially separated compartments within the microgels. This design concept allows for the introduction of a variety of individually triggered bioinspired behaviors, including protein capture, enzyme‐mediated content release, and stimuli‐triggered motility, each isolated in a distinct compartment enabling the use of the microgels as compartmentalized artificial cells. With this approach, the division of content and function seen in biological cells can be mirrored, which will underpin the generation of increasingly sophisticated and functional soft matter microdevices using bottom‐up synthetic biology principles.

## Graphical abstract

Artificial cells aim to replicate cellular form and function using bioinspired building blocks. In this article, a microfluidic technique is used to design spatially structured microgels for use as compartmentalized artificial cells, where the compartment size, composition, shape, and number are controllable. Additionally, the technique enables the insertion of functional organelles that demonstrate controlled release, directed motion and protein capture.

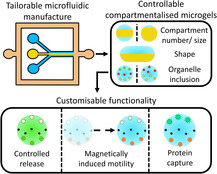

## 1 Introduction

Artificial cells are engineered constructs designed to replicate the structure, functionality, and behaviors of biological cells.^[^ ^1^, ^2^, ^3^ ^]^ They are typically constructed by assembling a variety of biological and biomimetic building blocks together, using bottom‐up synthetic biology principles.^[^ ^4^ ^]^ This strategy has enabled artificial cells to replicate cellular behaviors including motility,^[^ ^5^ ^]^ communication,^[^ ^6^ ^]^ compartmentalization,^[^ ^7^ ^]^ and signaling,^[^ ^8^ ^]^ allowing an increased understanding of cellular form and function^[^ ^9^ ^]^ and has led to the development of microdevices that can be used for a range of biotechnological applications, including drug delivery,^[^ ^10^ ^]^ biosensing,^[^ ^11^ ^]^ and as cell therapies.^[^ ^12^ ^]^ However, a considerable amount of research is still required to increase the sophistication of artificial cells in order to ultimately reconstruct a living system from non‐living building blocks^[^ ^13^ ^]^ and to translate current engineered functionalities toward real‐world applications.

Of the aforementioned cellular characteristics, significant efforts have been invested in replicating the compartmentalized nature of biological systems in particular.^[^ ^14^, ^15^ ^]^ Compartmentalization enables distinct specialized processes to be separated and localized to different cellular regions,^[^ ^16^ ^]^ provides the ability for incompatible chemical reactions to occur simultaneously without interference,^[^ ^17^ ^]^ and allows the functioning of signaling pathways, essential for intra and inter cellular communication.^[^ ^18^ ^]^ Moreover, the creation of compartmentalized systems provides a mechanism for multistage activation^[^ ^19^ ^]^ and an avenue for the production of spatially segregated microreactors,^[^ ^20^ ^]^ which can be used to create increasingly advanced drug delivery systems and biocatalytic systems.

Consequently, a range of artificial cell and tissue‐like systems using a variety of bioinspired building blocks have been developed to mimic this ubiquitous feature of biological systems. These include vesicle‐based artificial cells containing smaller vesicle subcompartments,^[^ ^21^ ^]^ lipid membrane bound coacervates,^[^ ^22^ ^]^ droplet interface bilayer based structures on cellular^[^ ^23^ ^]^ and tissue^[^ ^24^ ^]^ length scales, multicompartment polymeric vesicles,^[^ ^25^ ^]^ and capsosomes containing vesicle subcompartments.^[^ ^26^ ^]^

Hydrogels, 3D interconnected networks of hydrophilic polymers surrounded by an aqueous environment,^[^ ^27^ ^]^ have also emerged as an outstanding material for building multicompartment artificial cell systems.^[^ ^28^, ^29^, ^30^ ^]^ This is due to hydrogels possessing cell mimetic characteristics,^[^ ^31^ ^]^ being biocompatible,^[^ ^32^ ^]^ and having the ability to encapsulate a range of bioinspired and biological componentry such as lipid vesicles,^[^ ^33^ ^]^ magnetic particles,^[^ ^34^ ^]^ and bacteria.^[^ ^35^ ^]^ As a result, hydrogel structures have been produced, often through the use of droplet microfluidics, which can have spherical and biconcave shapes^[^ ^36^ ^]^ and can possess multiple compartments of tuneable sizes which can co‐ deliver multiple drugs.^[^ ^37^ ^]^ This has enabled a range of different hydrogel architectures to show biomimetic functionality. An example of this is the encapsulation of different enzymes within different hydrogel compartments, where the enzymes can then recreate multistep enzymatic pathways. The conditions required for each enzymatic reaction are different and incompatible with each other;^[^ ^30^ ^]^ without compartmentalization the multistep enzymatic pathway would not work. Another example involved hydrogels encapsulating a variety of coacervate microreactors, where each type of microreactor population was encapsulated in a different hydrogel block which could be interchanged, enabling a modular construction of a spatially separated multicompartment system.^[^ ^38^ ^]^ Similar functionality was also shown on the microscale.^[^ ^39^ ^]^ A final example demonstrated communication between colloidosome colonies within different hydrogel compartments and the hydrogel structure, by using different gelating strategies in the different hydrogel compartments; individual hydrogel compartments could be selectively degraded by the chemical reactions performed by the embedded colloidosome colonies.^[^ ^40^ ^]^

In the above examples, a variety of different hydrogels (polyethylene glycol, agarose, poly(*N*‐isopropylacrylamide, and alginate) and production techniques (droplet microfluidics, bulk hydrogel gelation, and droplet templated gelation) were employed to generate the biomimetic hydrogel structures, each designed to replicate a *single* biological function, e.g., control of shape, communication between different compartments or spatial separation. However, within cellular systems, a range of spatially segregated biomimetic functions are present.^[^ ^41^ ^]^ Consequently, there exists a need to design hydrogel systems where *multiple* biomimetic functionalities can be shown within a single chassis in order to improve the biomimicry and downstream functionality of hydrogel structures.

To address this need, we have developed a microfluidic methodology for generating cell‐sized monodisperse microscale two and three compartment biomimetic hydrogels in high throughput. The compartmentalized microgels are generated through the gelation of alginate,^[^ ^42^ ^]^ a biocompatible, naturally occurring polymer.^[^ ^43^ ^]^ The microfluidic production strategy enables us the tailor the size and composition of individual sub‐compartments, in addition to the overall shape and size of the microgels. We also demonstrate the ability to adjust the number of compartments present through the alteration of the microfluidic device, and to incorporate a range of functional organelles into the hydrogel compartments. The different populations of organelles are spatially separated from each other in each compartment and can be altered in a modular fashion to provide customizability over the function of each compartment. We finally demonstrate that the organelles embedded in each compartment can be activated independently, showing biomimetic behaviors including protein capture, controlled release, and stimuli driven motility. This range of behaviors makes our biomimetic microgels promising as a artificial cell chassis. We anticipate that the development of our versatile microfluidic technique ties together functionality which has existed in separate hydrogel microsystems before and will enable the creation of a variety of next‐generation compartmentalized hydrogel based artificial cells, in addition to programmable biomimetic soft matter microdevices.

## 2 Results and Discussion

### 2.1 Assembling Compartmentalized Biomimetic Microgels

**Figure 1:**
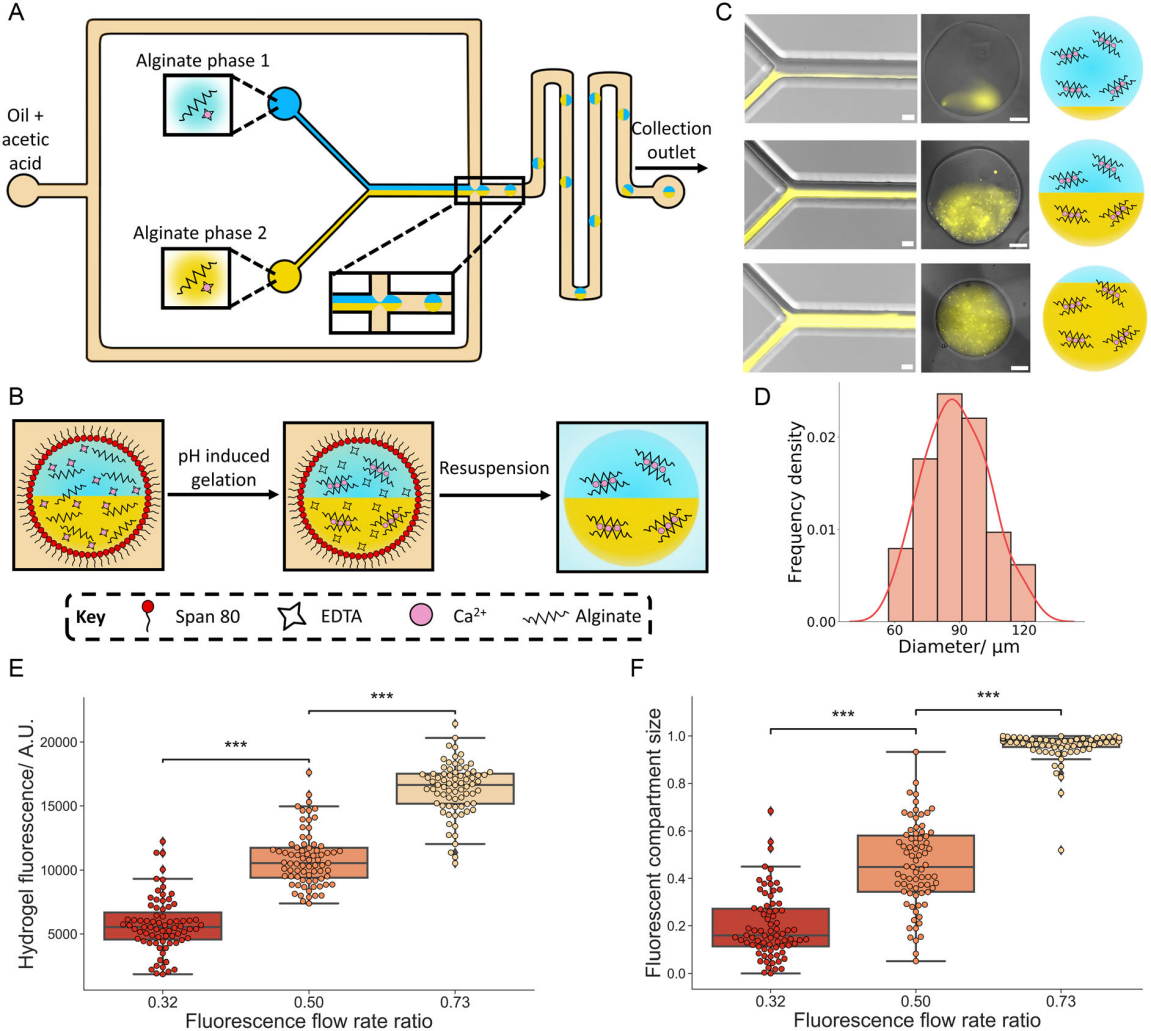
Assembly of two compartment microgels. A) An illustration demonstrating the production of two compartment microgels in a microfluidic device. Two different aqueous streams containing alginate meet and co‐flow until they meet an oil stream containing acetic acid at a flow focusing junction. This generates aqueous droplets with a compartment composition determined by the relative flow rates of the co‐flowing aqueous phases in the oil phase. The droplets are then gelated in flow by the surrounding acetic acid, and the two compartment microgels are collected upon exiting the device. B) A schematic showing the gelation procedure of the two compartment microgels. The EDTA‐Ca complex in the two compartment droplets dissociates upon a pH change produced by the acetic acid in the oil phase. This enables the free Ca^2+^ to cross‐link the alginate chains and gelate the droplets. The droplets are then resuspended in aqueous buffer where the compartmentalized microgels can be observed. C) Microscopy images and accompanying schematics of the production of two compartment microgels with a variety of compartment sizes. By varying the flow rate ratio between the two alginate containing solutions (with one containing fluorescently labelled alginate), a variety of different sized compartments can be generated in the two compartment microgels. The images show the production of two compartment microgels with a small fluorescent alginate compartment, two equal sized compartments, and a large fluorescent alginate compartment produced from 4 wt% alginate flow rate ratios of 0.19, 0.59, and 0.91, respectively. The microscopy images are generated from a fluorescence image of the rhodamine tagged alginate overlaid on a bright‐field image. The scale bars on the microfluidic images are 50 μm while the scale bars on the hydrogel images are 20 μm. D) Histogram with a kernel density estimation fit (red line) showing the average diameter of a population of two compartment microgels, the mean diameter was 88 μm and the polydispersity index was 0.028. The histogram was obtained from the analysis of *n* = 100 two compartment microgels. E) Box plots demonstrating how the fluorescence intensity of the microgels increases with a higher fluorescence flow rate ratio. The p values were $1.24\times 10^{-30}$ and $4.64\times 10^{-34}$. F) Box plots showing how the fluorescent compartment size within the microgels increases with a higher fluorescence flow rate ratio. The p values were $1.51\times 10^{-17}$ and $6.22\times 10^{-40}$. *n* = 75 hydrogels were analyzed for each box plot in panels E and F.

We generated the compartmentalized microgel chassis through microfluidics using an acid triggered gelation approach previously developed for cell encapsulation.^[^ ^42^ ^]^ (**Figure** **1**A,B and Videos S1, S2, Supporting Infomation). The polydimethylsiloxane (PDMS) microfluidic devices used to create the two compartment microgels had two aqueous inlets containing two different alginate solutions which met and co‐flowed together until meeting a faster flowing oil stream containing acetic acid and Span 80 at a flow focusing junction (Figure S1, Supporting Infomation). At the flow focusing junction, droplets containing both aqueous solutions were formed and stabilized by the Span 80 surfactant. The H^+^ ions present from the acetic acid in the oil phase then diffused into the aqueous droplets and lowered the pH. This led to the dissociation of the Ca‐EDTA complex present in the droplets and the subsequent binding of the free Ca^2+^ ions to the alginate, causing gelation and hydrogel formation.^[^ ^44^ ^]^ The gelation process was quick enough to enable hydrogel formation before the complete mixing of the different alginate phases within the droplets, thus enabling the formation of different compartments within the produced hydrogels.^[^ ^42^ ^]^ To confirm this, we added fluorescently labeled alginate into one of the aqueous streams, enabling the generated compartments to possess different fluorescent signals within the hydrogels. On imaging the produced aqueous droplets in oil (Figure S2, Supporting Infomation), a localization of fluorescent signal to one section of the droplet could be seen, indicating that the aqueous phases remained separate in the droplets where they gelate.

Further characterization was then performed by resuspending the gelated droplets into aqueous buffer. On imaging the two compartment microgels (Figures 1C and S3, Supporting Infomation), the fluorescent signal from one alginate phase could be seen to be predominantly localized within one section of the produced microgels. This matched the droplet result and confirmed the production of the two compartment microgels. However, when compared to the line profile across the two aqueous streams on the microfluidic device (Figure S4, Supporting Information), a smaller difference in fluorescence across the microgel line profile could be seen, indicating a degree of mixing between the alginate phases during gelation. This could potentially be reduced by altering the exit channel geometry.^[^ ^45^ ^]^

We then explored varying the flow rate ratio between the two aqueous streams (Figure 1C). It was seen that through altering the pressures supplied, the flow rate ratio between the two aqueous streams could be varied (Figure S5, Supporting Infomation). This enabled the size of the fluorescent compartment to be altered, from being present within a small region to encompassing the majority of the microgel. This demonstrated that the compartment size could be dictated and tailored by adjusting flow rates, with different architectures being made without changing the device itself. Moreover through using PDMS based microfluidics, the size of the two compartment microgels can be altered by adjusting the microfluidic device dimensions,^[^ ^46^ ^]^ vital for mimicry of different sized cells.

We additionally analyzed the produced two compartment microgels on a population level (Figures 1D–F and S3, S6, Supporting Information). A population of the two compartment microgels had a mean diameter of 88 μm and a polydispersity index (PDI) of 0.028, lower than 0.1, which is considered a threshold for a monodisperse population.^[^ ^47^ ^]^ We also observed that on increasing the flow rate of the fluorescent phase, the total fluorescence of the produced microgels increased in addition to the size of the fluorescent compartment, corroborating the microscopy images in Figure 1C. The variation in compartment size within each population was attributed to the different orientations of the hydrogels while imaging. This can be seen through taking Z stacks of the microgels where compartment sizes can be seen to vary slightly at different heights (Figure S7 and Video S3, Supporting Information). Therefore, the microfluidic production procedure can also create monodisperse microgel populations with a range of tailored compartment sizes, another important property required for the construction of highly controlled soft matter microdevices.

Finally, we investigated the stability of the microgels by storing them in a fridge for 5 months (Figure S8, Supporting Information). Upon imaging, we observed that the microgels remained intact, verifying that they could be stored for prolonged periods, crucial for potential long lifespan applications, for instance controlled drug delivery.^[^ ^48^ ^]^

### 2.2 Properties of Compartmentalized Biomimetic Microgels

After demonstrating the assembly of two compartment microgels, we explored changing the number of compartments, shape, and their composition. To increase the number of internal compartments, we added an additional channel to the microfluidic device (**Figures** **2**A and S1, Supporting Information). This provided an additional co‐flowing alginate solution to form three compartment microgels. Fluorescently labeled alginate was then added to an alginate solution (the central stream) to visualize microfluidic generation and compartment formation (Figures 2B and S3, S7, Supporting Information). It could be seen that the sizes of the three alginate solutions in the microfluidic device and the size of the compartments in the three‐compartment microgels could be altered by changing the flow rate ratio between the phases in the same manner as before, with a smaller fluorescent alginate stream again producing a smaller fluorescent compartment. The population distribution for the three compartment microgels (Figure 2C) (mean diameter 121 μm, PDI 0.029) was also similar to the two compartment microgels. Furthermore, on a population level, the total fluorescence of the produced microgels increased (Figure 2D) in addition to the size of the fluorescent compartment (Figure 2E) when the fluorescent flow rate ratio was increased, further verifying that three‐compartment microgel formation can be controlled. These results showed that the number of compartments generated in the biomimetic microgels could be increased by altering the microfluidic device geometry^[^ ^49^ ^]^ while still maintaining the same control over the microgel properties. This enables the controllable production of increasingly sophisticated compartmentalized microgel motifs.

Figure 2Properties of compartmentalized biomimetic microgels. A) An illustration depicting the production of three compartment microgels in a microfluidic device through the use of three aqueous streams containing alginate. B) Microscopy images and accompanying schematics of the production of three compartment microgels with two different fluorescent compartment sizes. The alteration of the flow rate ratios between the three alginate solutions (with one containing fluorescently labeled alginate) produced the different compartment sizes. The images show the production of three compartment microgels with different sized fluorescent compartments, the compartments are denoted by the dashed lines. The three compartment microgel with the smaller fluorescent compartment was produced from 4 wt% alginate with a fluorescent to non fluorescent flow rate ratio of 0.15 while the other microgel was produced with a flow rate ratio of 0.44. The microscopy images are generated from a fluorescence image of the rhodamine tagged alginate overlaid on a bright‐field image. The scale bars on the microfluidic images are 50 μm while the scale bars on the hydrogel images are 20 μm. C) Histogram with a kernel density estimation fit (blue line) showing the average diameter of a population of three compartment microgels, the mean diameter was 121 μm and the polydispersity index was 0.029. The histogram was obtained from the analysis of *n* = 100 three compartment microgels. D) Box plots showing how the fluorescence intensity of the three compartment microgels increases with a higher fluorescence flow rate ratio. The p value was $8.17\times 10^{-38}$. E) Box plots illustrating how the fluorescent compartment size within the three compartment microgels increases with a higher fluorescence flow rate ratio. The p value was $2.84\times 10^{-29}$. *n* = 75 hydrogels were analyzed for each box plot in panels D and E. F) Schematic and embedded microscopy images showing the generation of elongated two compartment microgels on chip, the microscopy images are of elongated water in oil droplets in the microfluidic channels and the exit chamber. The scale bars are 100 μm. G) A diagram with accompanying microscopy images showing a different elongated two compartment microgel. The fluorescence image shows the fluorescent compartment, and the dotted lines denote the hydrogel location in the images. The scale bars are 50 μm. H) Box plots showing the difference in aspect ratios between populations of elongated and spherical microgels. The *p* value was $1.62\times 10^{-37}$. The populations were generated from analyzing *n* = 75 hydrogels. I) An illustration showing the formation of two compartment microgels with compartments created from different alginate weight percentages. J) A fluorescent image demonstrating the successful formation of two compartment microgels with different weight percentage compartments. The fluorescent signal is from the higher weight percentage alginate compartment. The scale bar is 50 μm.

**Figure:**
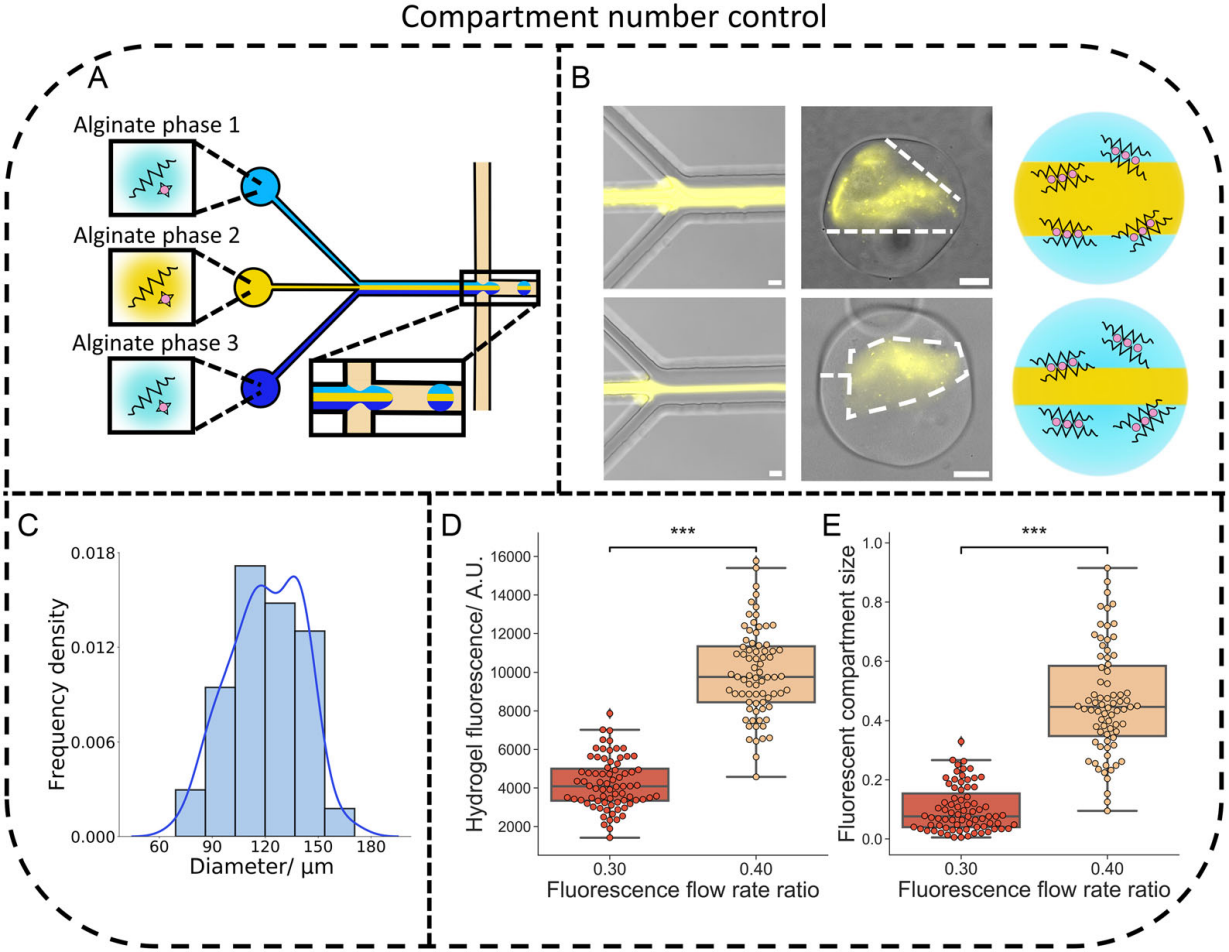

**Figure:**
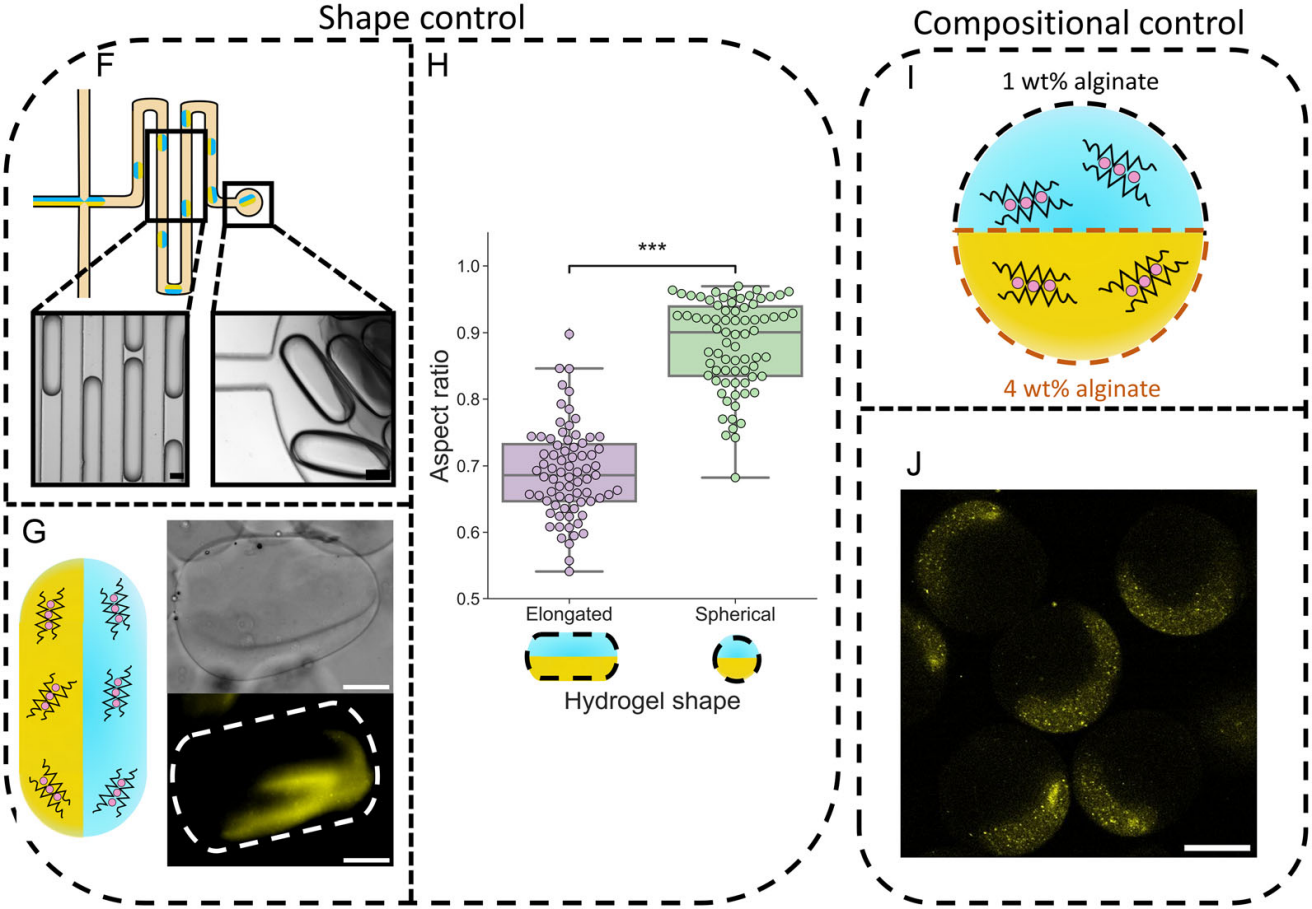

The shape of the compartmentalized microgels could also be controlled by increasing the size of the aqueous phase compared to the oil phase to form elongated aqueous plugs containing two alginate phases in the microfluidic device^[^ ^50^ ^]^ (Figure 2F). The plugs were then gelated within the microfluidic device channels and thus retained their shape on entering the exit chamber of the microfluidic device. Without gelation on chip, the plugs would deform into spherical droplets to minimize surface tension^[^ ^51^ ^]^ on exiting the device. On resuspending the plugs in an aqueous solution (Figures 2G and S3, Supporting Information), a localization of fluorescent signal to one portion of the plug could be seen, indicating that compartmentalized microgels of this geometry could be formed. To further contrast the geometry of the elongated plugs to the original microgels, the aspect ratio for each population was calculated^[^ ^52^ ^]^ (Figure 2H). The closer the value of the aspect ratio to 1, the more spherical the hydrogels. It could be seen that the elongated plugs possessed a smaller aspect ratio than the original microgels, further confirming that populations of elongated microgels could be generated. This demonstrated that compartmentalized microgels of different geometries could be generated, vital for mimicking cells with different shapes.^[^ ^53^ ^]^

Finally, the composition of the different compartments within the microgels was varied. This was achieved by using different weight percentages of alginate in the aqueous streams used to create the compartmentalized microgels. We initially showed that the microgels could be generated using different alginate weight percentages (Figure S9, Supporting Information) before generating a compartmentalized microgel with one compartment created from 1 wt% alginate and another created from 4 wt% alginate labelled with fluorescently labelled alginate (Figures 2I,J and S3, Supporting Information). Localization of the fluorescent signal could be seen, again indicating successful compartment formation, and verifying that the compartmentalized microgels could possess different alginate weight percentage compartment compositions. This enabled the compartments to possess different properties, for instance viscosities^[^ ^43^ ^]^ which differ in different cellular regions,^[^ ^54^ ^]^ therefore creating a more accurate multicompartment motif for cellular mimicking. Moreover different weight compositions of alginate can affect cell proliferation,^[^ ^55^ ^]^ indicating that through having different weight percentage compartments, the functionality of encapsulated substrates can be controlled in a compartment to compartment manner.

This approach provides multiple avenues to modify the properties of the compartmentalized microgels, potentially also in a combinatorial manner and systematically. By unlocking a broad compositional space, we enable exploration of a vast array of controllable, biomimetic compartmentalized configurations—essential for replicating a wide range of cellular systems, which is needed in artificial cell development. The technique is limited to feature sizes and channel widths larger than 1 μm^[^ ^56^ ^]^ and produced hydrogels of the same size order.^[^ ^57^ ^]^ However, as the compartment size and droplet size decreases, the more prominent the effects of mixing between the alginate streams on chip will become, leading to less defined compartments.

### 2.3 Inserting Organelles into Compartmentalized Biomimetic Microgels

**Figure 3:**
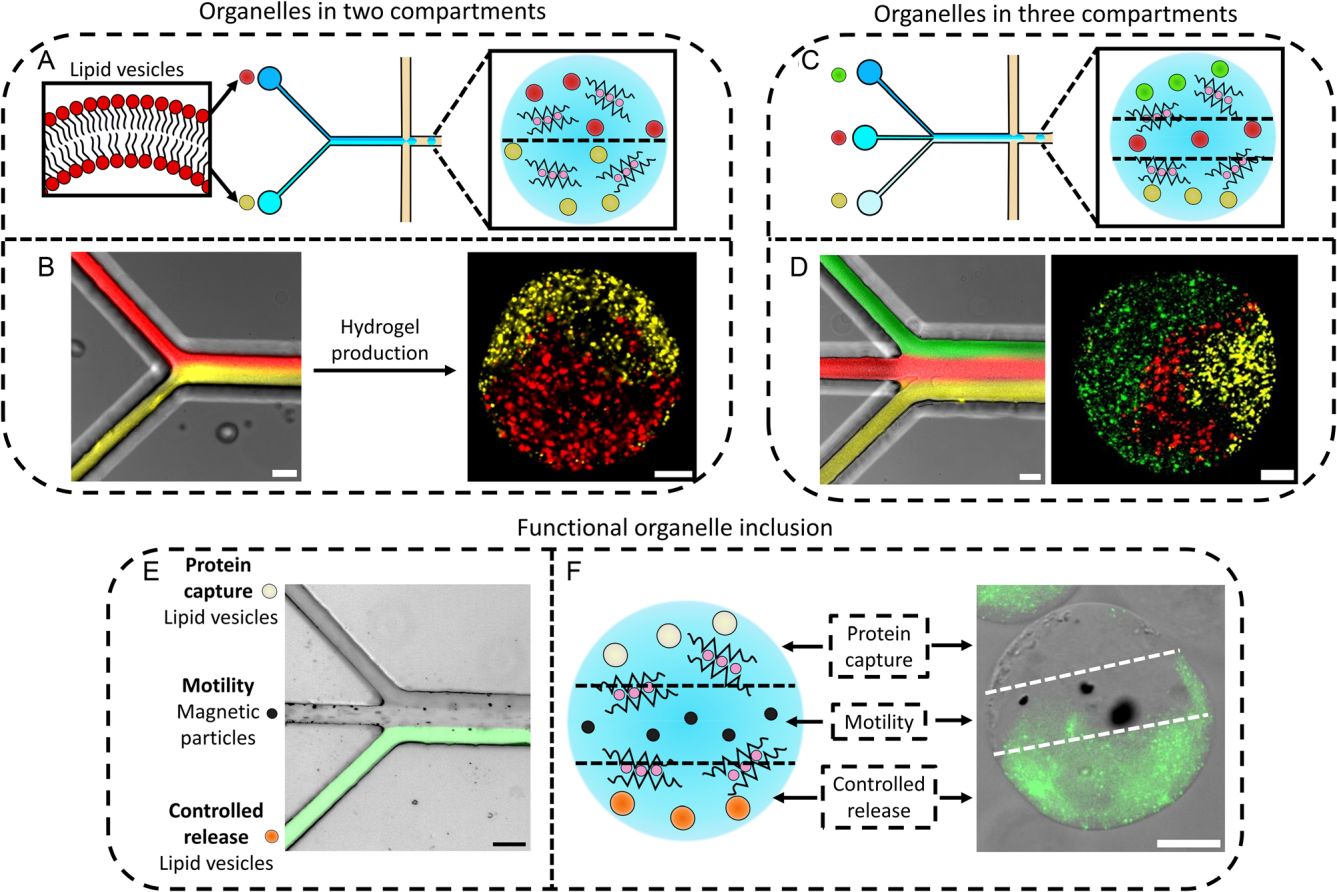
Organelle incorporation into compartmentalized microgels. A) Schematic showing the generation of two compartment microgels with organelles. Lipid vesicle organelles with different fluorescent dyes are placed into the two different alginate solutions which form the different compartments in the microgels, enabling organelles to be spatially separated within the biomimetic microgels. B) Microscopy images showing the generation of two compartment organelle containing microgels. The microfluidic image shows the fluorescent signals from the two different vesicle organelle populations overlaid on a bright‐field image, coflowing through the microfluidic device while the hydrogel image produced from overlaying the two fluorescent signals shows the separation of the two fluorescent organelle populations within the produced microgel. The microfluidic scale bar is 50 μm, and the hydrogel scale bar image is 20 μm. C) Illustration of the production of three compartment microgels with organelles through the use of three different fluorescent lipid vesicle populations in three different alginate solutions. D) Microscopy images showing the production of three compartment organelle containing microgels. The microfluidic image shows the fluorescent signals three different vesicle organelle populations overlaid on a bright‐field image, coflowing through the microfluidic device while the hydrogel image produced from overlaying the three fluorescent signals shows the separation of the three fluorescent organelle populations within the produced microgel. The microfluidic scale bar is 50 μm, and the hydrogel scale bar image is 20 μm. E) Microscopy image showing the production of three compartment microgels with different embedded organelles. These include lipid vesicles for protein capture, magnetic particles for motility, and lipid vesicles containing a fluorescent dye for controlled release. The fluorescent signal from the controlled release vesicles is overlaid on the bright‐field image to show the coflowing of the different organelle containing phases. The scale bar is 100 μm. F) Schematic with an accompanying microscopy image showing the separated functional organelles in a three‐compartment microgel. The overlaid fluorescent signal is from the fluorescent Calcein dye in the intact controlled release vesicles, and the dotted lines show the compartment boundaries. The scale bar is 20 μm.

After showcasing the properties and the tailorability of the compartmentalized microgels, we sought to increase the complexity of the produced structures by embedding organelles within the compartmentalized microgel chassis. This was performed through adding lipid vesicles, a previously used organelle mimic in hydrogels,^[^ ^33^ ^]^ (Figure S10, Supporting Information) with different fluorescent lipid labels into the alginate solutions used to create the compartmentalized microgels (**Figure** **3**A). In the two‐compartment microfluidic device, it could be seen that the fluorescent signals from the different organelle populations remained present and separated in the different coflowing alginate streams (Figure S11, Supporting Information). Again, a limited degree of mixing was seen between the streams, showing that at the interface between the two compartments, both organelle populations will be present. This interface region between the two fluorescence maxima was ≈20 μm. Within the produced microgels, the vesicle organelles were spatially separated as there was limited overlap between the regions of different fluorescent signal, though as with the microfluidic device, a small region of mixing was seen at the interface between the two compartments (Figures 3B and S12, Supporting Information).

Localization of lipid vesicle organelle populations into different hydrogel compartments was also successfully achieved in three compartment microgels (Figures 3C,D and S11, Supporting Information) and microgels with different morphologies (Figure S13, Supporting Information), confirming that insertion was compatible with the range of compartmentalized microgel motifs previously engineered and equipping the hydrogel structures with the ability to mimic the asymmetric spatial positioning of organelles in cells^[^ ^58^ ^]^ more closely than single compartment organelle containing hydrogels,^[^ ^33^, ^59^ ^]^ where the organelles are evenly distributed throughout the hydrogel chassis.

After verifying that organelle spatial positioning could be controlled in the compartmentalized microgels, the range of organelles inserted was expanded to include organelles that could mimic cellular functionality. These included magnetic particles that endowed the microgels with magnetically induced motility,^[^ ^60^ ^]^ lipid vesicles containing the small molecule dye Calcein (Figure S14, Supporting Information) that could be controllably released^[^ ^61^ ^]^ and lipid vesicles containing Biotin‐PE lipids which could capture proteins including Streptavidin.^[^ ^62^ ^]^ These were functionalities that have been demonstrated in isolation in hydrogel systems before,^[^ ^33^, ^63^, ^64^ ^]^ but not together in a spatially segregated microsystem. The organelle functions could be activated independently of each other or in tandem, enabling a range of biomimetic behaviors to be replicated in the microgel chassis. The organelles were selectively introduced into the compartmentalized microgels through the different alginate phases in a three‐component microfluidic device (Figure 3E and Video S4, Supporting Information) and could be seen to be present and spatially separated in the produced three compartment microgels (Figure 3F). The localization of fluorescent Calcein signal to a region of microgels shows that the vesicle organelles remained intact upon gelation. If the vesicles were bursting during hydrogel formation, no fluorescent localization would be observed as the small molecule dye would leak out of the microgel.^[^ ^33^ ^]^ This provided subcellular organization with a range of componentry, essential for the replication of complex cellular architectures and behaviors.

Furthermore, to confirm that the organelle containing compartmentalized microgels were stable and viable for utilization in a range of different environments, we placed the microgels in a range of different biological media (Figures S15 and S16, Supporting Information) where it was observed that the hydrogel chassis and the spatially separated organelle architecture remained intact. We also confirmed that the organelles within the microgels were stable and remained structurally intact for extended periods of time (5 months) in buffer (Figure S17, Supporting Information). Showing that the compartmentalized biomimetic microgels could be used to interact with biological cells^[^ ^65^ ^]^ and potentially have clinical applications,^[^ ^66^ ^]^ ideal for use as an artificial cell platform.

### 2.4 Activating Organelles in Three Compartment Biomimetic Microgels

After demonstrating that a variety of organelles could be spatially positioned in different compartments within the three compartment microgels shown in Figures 3E,F, the functional organelles (magnetic particles, Biotin‐PE functionalized lipid vesicles and vesicles containing Calcein) were activated with different stimuli to prove that the compartments could replicate biomimetic functionality in an orthogonal manner to each other.

**Figure 4:**
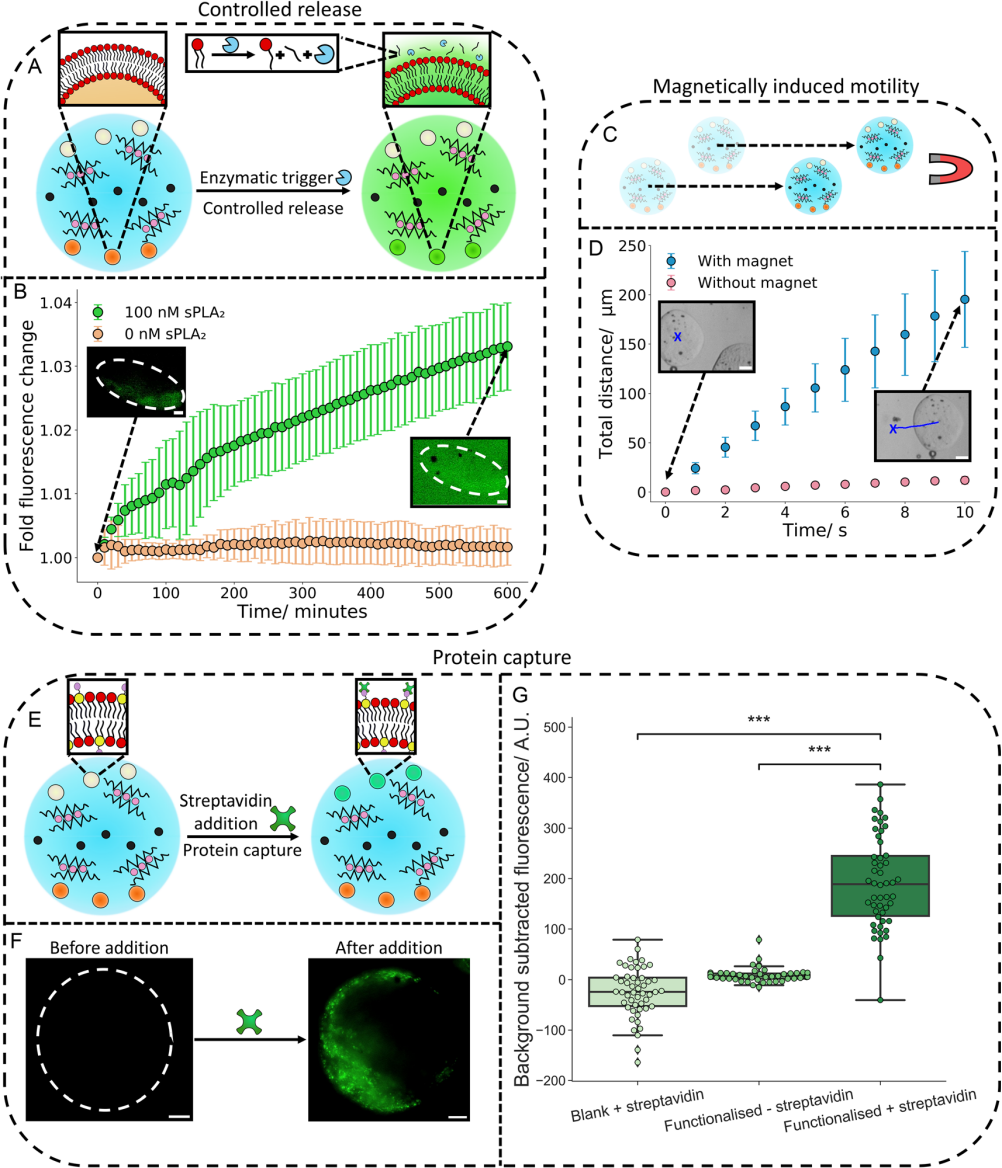
Three compartment microgel organelle functionality. A) An illustration of how controlled release is achieved from the vesicle organelles in the three compartment microgels. The addition of the enzyme sPLA_2_ (the blue partial circle) causes cleavage of lipid tails. This produces defects in the vesicle organelle bilayers causing the release of the entrapped Calcein dye. B) A time lapse graph showing on sPLA_2_ addition an increase in fluorescent signal is observed due to Calcein release from the vesicle organelles. There is no release without sPLA_2_ addition. The microscopy images show the fluorescence change between 0 and 600 min around a microgel exposed to sPLA_2_.The dotted circle shows the hydrogel position. The error bars represent the standard deviations from *n* = 3 microgel populations. The scale bars are 20 μm. C) A schematic illustrating the motion of the three compartment microgels on magnetic field application. D) Plot showing the movement of three compartment microgels over 10 s with and without magnetic field application. Motion is observed on magnetic field application. The embedded microscopy images show the movement of a microgel before and after magnetic field exposure. The X is the starting position, and the blue line showcases the trajectory taken in the 10 s. The error bars on the graph are the standard deviation of the motion of *n* = 10 three compartment microgels. The scale bars are 50 μm. E) A diagram showing how protein capture is achieved. On incubation with Streptavidin, the Biotin‐PE lipid (yellow lipid) present on the vesicle organelles will bind Streptavidin, generating a localized fluorescent signal on the organelles. F) Fluorescence images before and after fluorescent Streptavidin addition. On fluorescent Streptavidin addition, a localized fluorescent signal is observed indicating protein capture. The dotted circle shows the position of a hydrogel before Streptavidin addition. The scale bars are 20 μm. G) Box plots demonstrating how the fluorescence of microgels containing Biotin‐PE vesicles increases on fluorescent Streptavidin addition. The *p* values were $6.40\times 10^{-24}$ and $3.47\times 10^{-19}$. *n* = 50 hydrogels were analyzed for each condition.

The first spatially separated organelle we activated was the Calcein containing lipid vesicles. This was achieved through the addition of phospholipase A2 (sPLA_2_), an enzyme that cleaves lipid tails at the *sn‐*2 position, generating a fatty acid and a lyso‐phosphatidylcholine lipid in the vesicle membrane.^[^ ^67^ ^]^ This also leads to an increase in membrane permeability and the release of self‐quenched Calcein from within the vesicles and into the surrounding solution, causing an increase in fluorescence^[^ ^68^ ^]^ (**Figure** **4**A). The enzyme was smaller than the pore size of the hydrogels and so could readily diffuse to reach the vesicle organelles containing Calcein.^[^ ^33^ ^]^

On addition, the sPLA_2_ enzyme led to an increase in fluorescence and a delocalization of fluorescent signal from the controlled release organelle containing compartment of the microgel (Figure 4B and Video S5, Supporting Infomation). Without sPLA_2_, no release was observed. This demonstrated that this organelle population could be activated by an enzyme to controllably release cargo within the three compartment microgels.

The second organelle triggered within the three compartment microgels were magnetic particles. This was performed by positioning a magnet next to the three compartment microgels where the magnet would cause microgel migration toward the magnet (Figure 4C). On placing the magnet in close proximity to the microgels, directional microgel migration was seen toward the magnet while without magnet application, limited motion was observed (Figures 4D and S18; Video S6, Supporting Information). In both scenarios, the magnetic particles were seen to be stably entrapped in the hydrogel network. This demonstrated that a second organelle within the three compartment microgels could be activated independently to endow a biomimetic functionality.

Finally, we utilized the third Biotin‐PE functionalized vesicle organelle population to capture the protein Streptavidin, a protein smaller than the pore size of the hydrogels^[^ ^69^ ^]^ and thus could freely diffuse into the hydrogel structure. This was performed by adding 2 μM of fluorescently labeled Streptavidin to a solution containing the three compartment microgels (Figure 4E). On addition, the fluorescently labeled Streptavidin bound to the Biotin‐PE vesicle organelles and produced a localized fluorescent signal (Figure 4F). In the absence of organelles, the fluorescently labeled Streptavidin diffused through the hydrogels without any localization (Figure S19, Supporting Information) showing that the localization of signal was occurring due to the presence of the Biotin‐PE containing organelles. Moreover, it was observed that the fluorescence signal from the Streptavidin was stronger than the quenched Calcein (Figure S20, Supporting Information), attributing the change in fluorescence to the Streptavidin addition. This could be seen on a population level (Figure 4G) by the microgels with Biotin‐PE vesicles and Streptavidin possessing a larger localized fluorescent signal than vesicle functionalized microgels without Streptavidin and blank hydrogels with Streptavidin. The results therefore showed that the Biotin‐PE functionalized functionalized vesicle organelles within the three compartment microgels can be utilized for protein capture which could be triggered independently of the other aforementioned biomimetic functionalities.

The above results demonstrate that three different bioinspired organelles, present in different compartments can be activated independently to each other and on demand within the three compartment microgels to recreate biomimetic behavior. The spatial separation also enables the compartments to possess different properties (e.g., different local pH's), which may be incompatible to different organelle populations^[^ ^30^ ^]^ and enables the functionalities in each compartment to not interfere with each other.^[^ ^70^ ^]^ This expands the use of the three compartment microgels to devices that can replicate cellular behavior as well as the architecture, showing that the microgels can be used for a range of artificial cell functions. This is a key step toward replicating the complexity present in three compartment biosystems and again showcases the utility of the three compartment microgels.

## 3 Conclusions

In conclusion, we have developed a microfluidic manufacturing technique for microscale compartmentalized microgels, which are amenable for use as artificial cells. The number of compartments, compartment properties, and compartment size can be tailored along with the overall shape of the microgels, enabling the microgels to mimic the morphology and compartmentalization of a variety of cell types. Moreover, synthetic organelles possessing a variety of bioinspired functionalities can be positioned within the different compartments, replicating subcellular organization. Within the microgel chassis, we showed the different organelle populations were able to be independently activated to capture proteins, controllably release cargo, and perform stimuli triggered motility, providing a foundation to generate increasingly complex compartmentalized cell mimics. This foundation could be expanded upon by inserting active biological componentry into the generated compartments through optimizing the acid triggered gelation procedure.^[^ ^42^ ^]^ This would enable the development of multicompartment biohybrid systems and microscale spatially separated organism communities^[^ ^71^ ^]^ to further probe biological interactivity. Further studies could also be performed on assessing the potential use of the compartmentalized microgels in vivo, paving the way for their potential use in clinical applications^[^ ^72^ ^]^ due to the ability of alginate hydrogels to be implanted^[^ ^73^ ^]^ and the previous success of alginate microgels as drug delivery vehicles.^[^ ^74^ ^]^ Alternatively, the three compartment microgels could be engineered to include cascade reactions, providing interactivity between the compartments^[^ ^75^ ^]^ to mimic enzymatic pathways,^[^ ^76^ ^]^ in addition to avenues for exploring the mechanism behind communication between the compartments in more detail. This could enable multistep drug synthesis,^[^ ^77^ ^]^ crucial for the development of advanced drug delivery platforms and biocatalysts.

## 4 Experimental Section

4.1

4.1.1

##### Materials

Sodium alginate was purchased from Sigma Aldrich (Gillingham, UK). The fluorescent Rhodamine B labelled alginate was purchased from HAworks (New Jersey, USA). Both types of alginate were used without further purification. The lipids 1‐palmitoyl‐2‐oleoyl‐glycero‐3‐phosphocholine (POPC) and 1,2‐dioleoyl‐sn‐glycero‐3‐phosphocholine (DOPC) were purchased from Avanti Polar Lipids (Alabaster, AL) as powders and used without further purification. The lipids 1,2‐dioleoyl‐sn‐glycero‐3‐phosphoethanolamine‐N‐(Cyanine 5) (Cy5‐PE), 1,2‐dioleoyl‐sn‐glycero‐3‐phosphoethanolamine‐N‐(lissamine rhodamine B sulfonyl) (ammonium salt) (Rh‐PE), 1,2‐dioleoyl‐sn‐glycero‐3‐phosphoethanolamine‐N‐(7‐nitro‐2‐1,3‐benzoxadiazol‐4‐yl) (ammonium salt) (NBD‐PE), and 1,2‐dioleoyl‐sn‐glycero‐3‐phosphoethanolamine‐N‐(biotinyl) (sodium salt) (Biotin‐PE) were purchased from Avanti Polar Lipids (Alabaster, AL) as chloroform stocks. The MagneHis Ni‐Particles were obtained from Promega (Southampton, UK). PDMS Sylgard 184 elastomer kits were purchased from Dow Corning (Michigan, USA). The Streptavidin, Alexa Fluor 488 conjugate, Dulbecco's‐modified eagle medium (DMEM) with high glucose and pyruvate, Roswell Park Memorial Institute (RPMI) 1640 Medium with no glutamine and no phenol red, and Fetal Bovine Serum were purchased from ThermoFisher Scientific (UK). The remaining reagents which included chloroform, ethylenediaminetetraacetic acid (EDTA), Sorbitan monooleate (Span 80), mineral oil, calcium chloride, HEPES buffer, Sephadex G‐50, sucrose, acetic acid, potassium chloride (KCl), Triton‐X100, Calcein dye, and the secretory phospholipase A2 (sPLA2) from honeybee venom (Apis mellifera) were all obtained from Sigma Aldrich (Gillingham, UK) unless explicitly mentioned.

##### Vesicle Production

To produce the small unilamellar vesicles, lipid powders were initially dissolved in chloroform solution to make stock solutions. Appropriate amounts of the stock solutions were then combined to generate solutions of the appropriate compositions and weights that would be used to produce lipid films. All lipid films had the final weight of 5 mg, the fluorescent lipid dyes (Cy5‐PE, Rh‐PE, NBD‐PE) were added at a concentration of 0.3 mol%, and the Biotin‐PE lipid was used at a concentration of 1 mol%. The fluorescent lipid dyes were combined with DOPC lipids, while POPC lipids were used with Biotin‐PE or for Calcein encapsulation. The solutions were then mixed for 1 min before gentle chloroform evaporation under a stream of N_2_ to produce a lipid film. The lipid film was then further dried overnight under vacuum to remove any residual chloroform. The dried lipid films were rehydrated with 0.5 m sucrose and 100 mM KCl for experiments not requiring Calcein encapsulation. For Calcein encapsulation, the lipid films were rehydrated with 50 mM Calcein, 100 mM HEPES, and 100 mM KCl. The final lipid concentration of all the hydrated lipid films was 10 mg mL^−1^. The hydrated lipid films were then freeze–thawed 5 times. This involved flash freezing the hydrated lipids in liquid N_2_ before heating the sample to 50 °C and vortexing for 30 s. After the freeze–thaw cycles, the lipid solution was extruded through 0.1 μm polycarbonate membranes 21 times to produce a vesicle population of ≈146 nm in size (Figure S10, Supporting Infomation). The Calcein‐filled vesicles were then passed through a size exclusion column containing Sephadex G‐50 with an eluent of 0.5 M sucrose and 100 mM KCl. About 200 μL of the Calcein filled vesicles were added to the column, and the purified vesicles were collected in fractions of 300 μL. All vesicles were used within 2 days to minimize cargo leakage and vesicle aggregation.

##### Microfluidic Device Production

The PDMS microfluidic devices were produced by initially generating a design on a silicon wafer (Inseto). The patterned wafers were created by depositing a photoresist (SU‐8 3050, Kayaku Advanced Materials, MA, USA) of a depth of 100 μm using a spin coater. The wafers were then baked and developed by exposing the wafer to UV light (365 nm, 300 mJ cm^−2^) through an acetate photomask (Micro Lithography services, UK) that contained the device design. The wafers were then baked again before the unexposed features were removed using propylene glycol monomethyl ether acetate developer and were rinsed with isopropyl alcohol. Finally, the patterned wafers were silanized with trichloro(1H, 1H, 2H, 2H‐perfluorooctyl)silane under vacuum overnight.

The produced patterned wafers then had degassed PDMS (10:1 Elastomer: Curing agent) poured over them. The PDMS was then left to cure for at least 3 h at 60 °C. The cured PDMS with the embedded microfluidic design was then removed from the wafer before 1.5 mm holes were punched into the inlet and outlet ports on the design. The PDMS with the device design was finally irreversibly bonded to a glass slide through exposing the glass slide and the patterned side of the PDMS to plasma (Harrick Plasma, NY, USA) for 1 min and 30 s before contacting the surfaces together to form an irreversible bond between the PDMS and glass slide. The assembled devices were then left overnight to ensure complete bonding before use.

##### Compartmentalized Biomimetic Microgel Manufacture

Stock aqueous solutions containing alginate (0.5–4 wt%), 0.5 M Sucrose, and 50 mM of a Ca‐EDTA complex were prepared, and 4 wt% alginate was typically used unless specified explicitly. When lipid vesicles organelles were required to be included into the alginate solutions, an alginate stock solution containing double the alginate concentration required, 0.5 M sucrose and 100 mM Ca‐EDTA were diluted in half using the lipid vesicle organelles. This generated a final stock solution containing the required wt% of alginate, lipid vesicle organelles, 0.5 M sucrose, 50 mM Ca‐EDTA, and 50 mM KCl. For experiments using fluorescent Rhodamine B tagged alginate, the dye was added at 0.1 wt% to the alginate stock solutions. When magnetic particles were used as organelles, the magnetic particles were added at a concentration of 10 mg mL^−1^ to the alginate stock solutions, this was a 10‐fold dilution of the purchased magnetic particle stock. The oil phase used in the microfluidics was produced by adding 1 V V^−1^% acetic acid and 5 wt% Span 80 into mineral oil.

The aqueous alginate solutions and oil were then flowed into the appropriate inlets in the PDMS microfluidic device (Figure S1, Supporting Infomation). Polyethylene tubing (Kinesis, UK) was used to deliver solutions both toward and from the microfluidic chip, and a pressure pump (Elveflow, Paris, France) was used to control the flow rates of the different aqueous and oil phases. The aqueous phases coflow together within the device before forming aqueous hydrogel precursor droplets at an aqueous/oil flow focusing junction. The oil phase flow rate was larger than the flow rates of the aqueous phases to ensure droplet formation occurred at the aqueous/oil flow focusing junction. The flow rates between the aqueous phases were varied to alter the sizes of compartments within the microgels.

The pressure ratios were calculated using Equation (1), where $P_{f}$ was the pressure supplied to the fluorescent aqueous stream and $P_{\text{nf}}$ was the pressure supplied to the nonfluorescent aqueous stream.

(1) $$\text{Pressure ratio}=\frac{P_{f}}{P_{\text{nf}}}$$

The aqueous flow rate ratios were calculated using Equation (2), where $Q_{f}$ was the size of the fluorescent aqueous stream and $Q_{t}$ was the size of the total (fluorescent and nonfluorescent) aqueous streams.

(2) $$\frac{\text{Fluorescent flow rate}}{\text{Total flow rate}}=\frac{Q_{f}}{Q_{t}}$$

The produced compartmentalized microgels were collected by connecting outlet tubing from the microfluidic device to an Eppendorf tube. The Eppendorf tub containing the compartmentalized microgels and oil phase was then centrifuged for 5 min at 9000×g to produce a pellet. The supernatant consisting of the oil phase was then removed before the pellet could be stored in a fridge. To perform experiments with the compartmentalized microgels, they were resuspended in buffer containing 0.5 M sucrose, 100 mM HEPES (pH 7.4), 100 mM KCl, and 20 mM CaCl_2_. The addition of the CaCl_2_ prevented any unwanted hydrogel degradation occurring. For any experiments conducted in a different media, the compartmentalized microgels in buffer were diluted in a 1:20 ratio into the desired media.

##### Dynamic Light Scattering

To size the extruded vesicles, a Malvern Zetasizer Ultra instrument (Malvern, UK) with a 632.8 nm HeNe gas monochromatic laser was used. Back scattered light was detected at an angle of 173° from the transmitted beam that minimized unwanted reflection. The extruded vesicles were analyzed by diluting the vesicles in a 1:10 ratio in sucrose buffer (0.5 M sucrose, 100 mM HEPES (pH 7.4), 100 mM KCl, and 20 mM CaCl_2_).

##### Widefield Microscopy

A Nikon eclipse Ti2‐U inverted microscope with a CoolLED pE‐300^white^, a 4× objective, 10× objective, 20× objective with a 1.5× magnification applied or a 100× objective and a Nikon DS‐Qi2 camera were used to image and record time lapses of the microfluidic devices and compartmentalized microgels. Fluorescence images/time lapses were collected using either a TRITC‐B, GFP, or FITC filter cube.

To image/record time lapses with the compartmentalized microgels, the hydrogels were imaged using PDMS wells on a cover slip. The sample wells were sealed by placing another cover slip on top of the sample well. For the organelle capture/activation experiments, fluorescent Streptavidin/ sPLA_2_ were added to the sample in buffer immediately prior to imaging.

For all comparative population analysis, statistical analysis was performed using the SciPy.stats library in python where an unpaired *t*‐test with unequal sample variances was used. The *p* values were set to the following levels: * < 0.05, ** < 0.01, and *** < 0.001.

Line profile intensities obtained from microscopy were normalized using Equation (3). In the equation, $F_{\text{max}}$ was the maximum fluorescence intensity and $F_{x}$ was the fluorescence intensity at a given point on the line profile.

(3) $$\text{Normalised intensity}=\frac{F_{x}}{F_{\text{max}}}$$

To compare different length line profiles, the distances of the line profiles were normalized using Equation (4) where $d_{\text{max}}$ was the maximum distance and $d_{x}$ was the distance at a given point on the line profile.

(4) $$\text{Normalised distance}=\frac{d_{x}}{d_{\text{max}}}$$

Equation (5) was used to calculate the PDI of the compartmentalized microgels. This was obtained through using the standard deviation (*σ*) and mean (*d*) of the population analyzed.^[^ ^78^ ^]^

(5) $$\text{PDI}={\left(\frac{\sigma }{d}\right)}^{2}$$

The aspect ratios for the elongated and spherical microgels were calculated using the below equation. ${\text{Feret diameter}}_{\text{min}}$ was the minimum Feret diameter, and ${\text{feret diameter}}_{\text{max}}$ was the maximum Feret diameter.

(6) $$\text{Aspect ratio}=\frac{{\text{feret diameter}}_{\text{min}}}{{\text{feret diameter}}_{\text{max}}}$$

When line profiles from different fluorescent channels were required to be compared directly across a line, Equation (7) was used. $F_{\text{max}}$ was the maximum fluorescence intensity, $F_{\text{min}}$ was the minimum fluorescence intensity, and $F_{x}$ was the fluorescence intensity at a given point on the line profile.

(7) $$\text{Normalised channel intensity}=\frac{F_{x}-F_{\text{min}}}{F_{\text{max}}-F_{\text{min}}}$$

Equation (8) was used to normalize the diameter of the compartmentalized microgels over time. $D_{I}$ was the initial diameter, and $D_{t}$ was the diameter at a given time point.

(8) $$\text{Normalised diameter}=\frac{D_{t}}{D_{I}}$$

Equation (9) was used to analyze the release time lapses through calculating the fold fluorescence change. $F_{t}$ corresponded to the fluorescence intensity at a given time point and $F_{0}$ indicated the initial fluorescence intensity.

(9) $$\text{Fold fluorescence change}=\frac{F_{t}}{F_{0}}$$

Equation (10) was used to calculate the background subtracted fluorescence. *F* is the average fluorescence intensity of the region analyzed, while $F_{B}$ is the average fluorescence intensity of a nearby background region.

(10) $$\text{Background subtracted fluorescence}=F-F_{B}$$

##### Confocal Microscopy

For confocal imaging of the produced compartmentalized microgels, a Leica stellaris 8 was used with 10× and 20× objectives and a WLL (white light) laser. The emission was recorded using HyD detectors, and the pinhole was set to a diameter of 1 Airy unit. The lasers excited the relevant fluorophores at their excitation maxima and the detectors recorded emission at their emission maxima. For the fluorophores detected with the confocal microscope, these values were 460/535 nm (NBD‐PE), 546/567 (Rhodamine‐B), 560/583 nm (Rh‐PE), and 639/663 (Cy5‐PE).

The obtained images and Z stacks were used to calculate the fluorescence intensity of the microgels and the compartment sizes. The compartment sizes were obtained by thresholding fluorescent hydrogel images into binary images where one color corresponded to a fluorescent compartment and the other color to a nonfluorescent compartment. The size of the fluorescent compartment compared to the total hydrogel was then calculated to obtain the compartment size in the hydrogel. The thresholding procedure is detailed more in Figure S6, Supporting Infomation.

##### Organelle Triggered Magnetic Motility

To perform the magnetic particle organelle motility experiments, sample wells made from 2 mm hard continuous poly(methyl methacrylate) (Clarex 001, Weatherall Equipment and Instruments Ltd) with double‐sided acrylic adhesive were produced using a laser cutter (VLS 2.30 Universal Laser Systems). The cut wells were adhered to a glass slide before Rain‐X was applied to increase the hydrophobicity of the glass slide and limit microgel adherence. To induce magnetic motion, an ultrahigh performance N52 Neodymium magnet (50 mm diameter, 10 mm thickness) (Magnet Expert) was positioned at a distance of 7 mm from the center of the cut wells.

For recording bright‐field videos of the magnetically induced motion, a Nikon TE2000‐U inverted microscope with a Ximea MQ013MG‐E2 camera and 4× objective was used. The videos were then analyzed with the manual tracking tool on ImageJ to select the center of the compartmentalized microgels at 1 s intervals to extract *X* and *Y* coordinates. The *X* and *Y* coordinates were then inputted into Equation (11) to calculate the total distance moved. In the equation, *X* and *Y* are the *x* and *y* coordinates of the hydrogel, *n* is the number of time steps analyzed, and *i* is the current time step.

(11) $$\text{Total distance}=\sum_{i=1}^{n}\sqrt{{\left(X_{i+1}-X_{i}\right)}^{2}+{\left(Y_{i+1}-Y_{i}\right)}^{2}}$$

## Conflict of Interest

The authors declare no conflict of interest.

## Author Contributions

**Matthew E. Allen**: conceptualization (lead); data curation (lead); formal analysis (lead); investigation (lead); methodology (lead); visualization (lead); and writing—original draft (lead), **James W. Hindley**: project administration (supporting); supervision (supporting); and writing—review and editing (supporting), **Robert V. Law**: project administration (supporting); supervision (supporting); and writing—review and editing (supporting), **Oscar Ces**: funding acquisition (equal); project administration (equal); supervision (equal); and writing—review and editing (supporting), and **Yuval Elani**: conceptualization (lead); funding acquisition (lead); supervision (lead); and writing—review and editing (lead). **Matthew E. Allen**: designed and performed the experiments, analyzed the data, and wrote the manuscript. **Robert V. Law**: helped revise the manuscript. **Yuval Elani**, **Oscar Ces**, and **James W. Hindley**: designed experiments, revised the manuscript, and supervised the project.

## Supporting information

Supplementary Material

## Data Availability

The data that support the findings of this study are available from the corresponding author upon reasonable request.
