# Supplementary material for: Microfluidic Production of Spatially Structured Biomimetic Microgels as Compartmentalized Artificial Cells
Source: Small Sci. 2025 Feb 6;5(4):2400320. doi: 10.1002/smsc.202400320 (PMC12245075; doi:10.1002/smsc.202400320)
Supplement: Supplementary file 1 — Supplementary Material [file SMSC-5-2400320-s001.zip › smsc202400320-sup-0001-SuppData-S1.pdf]

Supporting Information

Title: Microfluidic Production of Spatially Structured Biomimetic Microgels as Compartmentalised Artificial Cells

Matthew E. Allen[1,2,3,4], *James W. Hindley*[1,2,4], *Robert V. Law*[1,2,4], *Oscar Ces*[1,2,4], *Yuval Elani*[2,3,4]*

[1]Department of Chemistry, Imperial College London, Molecular Sciences Research Hub, 82 Wood Lane, London, W12 0BZ
[2]Institute of Chemical Biology, Imperial College London, Molecular Sciences Research Hub, 82 Wood Lane, London, W12 0BZ
[3]Department of Chemical Engineering, Exhibition Road, Imperial College London, South Kensington, London SW7 2AZ
[4]FabriCELL, Imperial College London, Molecular Sciences Research Hub, 82 Wood Lane, London, W12 0BZ

E-mail: y.elani@imperial.ac.uk

Supporting figures

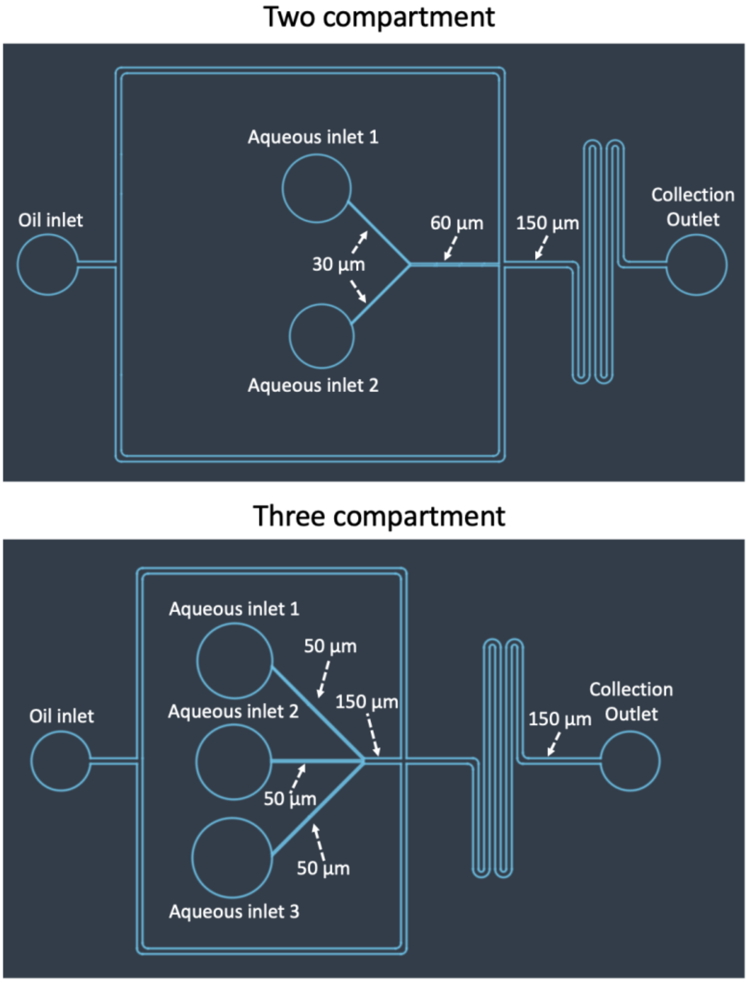

Figure S1: Schematics of the microfluidic devices. The annotations indicate the widths of the different channels and the different outlets and inlets in the two and three compartment microfluidic devices. The channel depth was always 100 µm.

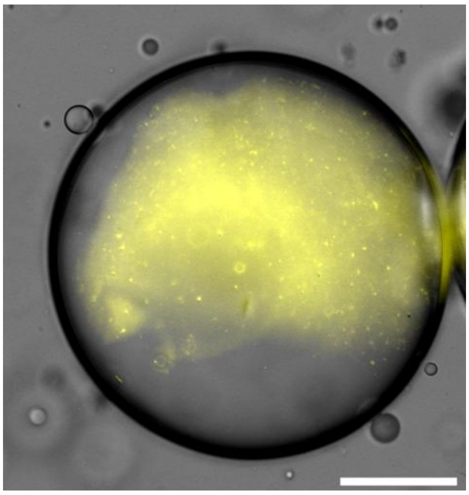

Figure S2: Two-compartment hydrogel formation in water in oil droplets. A merged fluorescence and brightfield image showing hydrogel compartment formation in the generated water in oil droplets through the microfluidic chip. The scale bar is 50 µm.

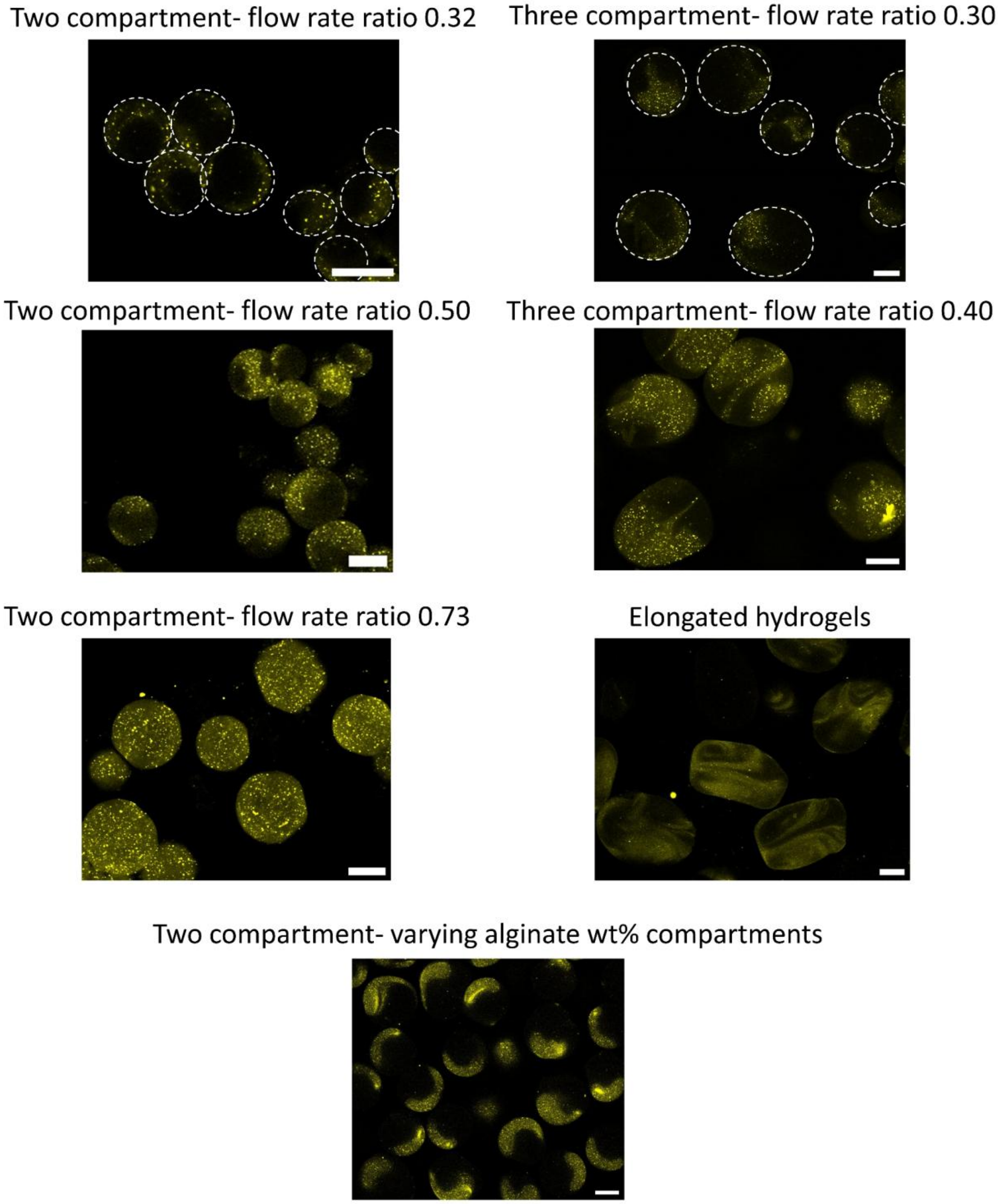

Figure S3: Population images of compartmentalised microgels. Confocal fluorescence population images of microgels used for analysis throughout figures 1 and 2. The dotted lines show the positions of the microgels with a small fluorescent compartment. The differences in microgel compartment size arise from the orientations of the hydrogels. The varying wt% compartment image is a wider field of view of the image used in figure 2J. The scale bars are all 50 µm.

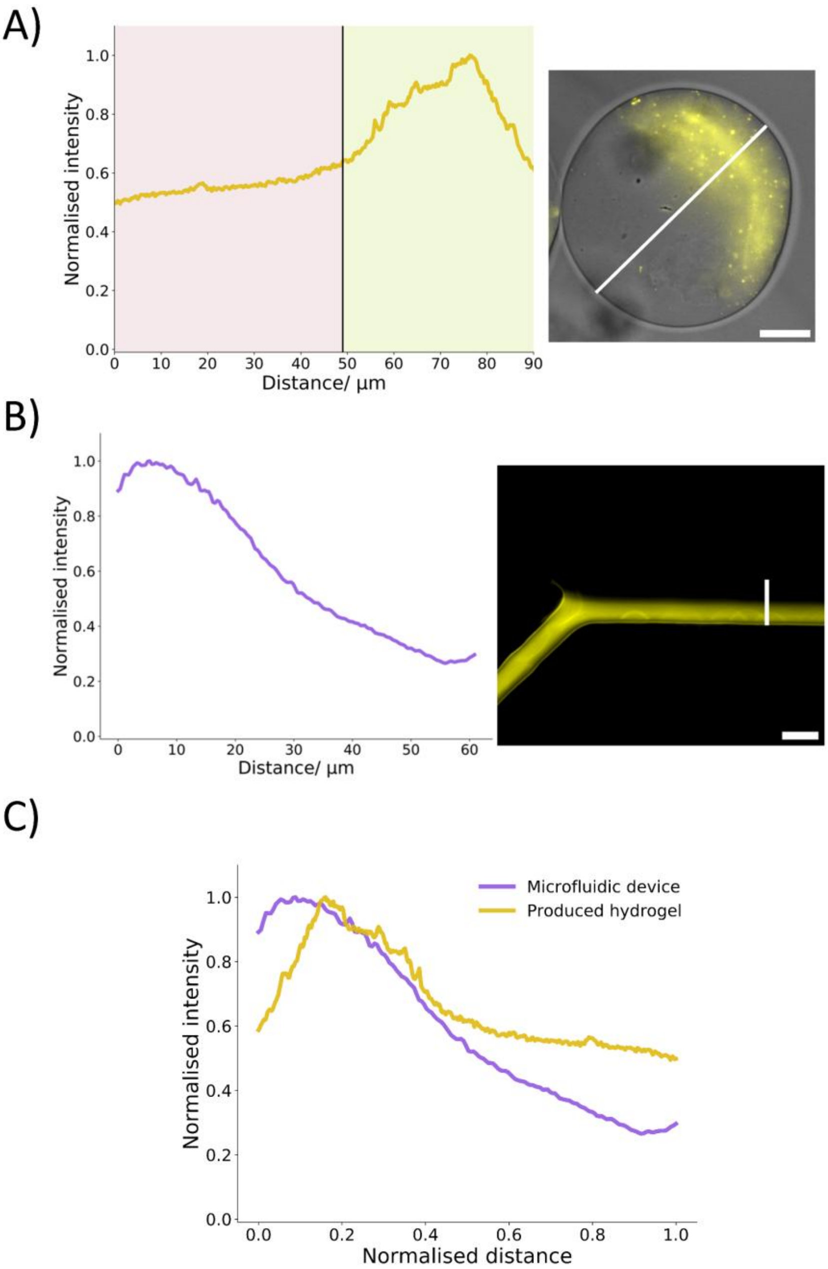

Figure S4: Comparing line profiles on chip and in the produced two compartment microgels. A) The line profile across a two compartment microgel. The fluorescent alginate signal can be seen to more pronounced within one compartment (green region on the line profile plot) and shows that there are two distinct compartments within the microgels generated using two aqueous solutions on chip. The line profile was extracted from the white line on the accompanying microscopy image which is a fluorescence image overlaid on a brightfield image. The scale bar is 20 µm. **B)** The line profile across two aqueous streams in a microfluidic device. The fluorescent alginate signal decreases over the aqueous channel which houses two aqueous coflowing streams. This shows that the fluorescent signal is localised to one stream but diffusion into the second stream is visible. The line profile was extracted from the white line on the accompanying fluorescence microscopy image. The scale bar is 50 µm. **C)** A graph comparing the plotted line profiles from panels A and B. The produced two compartment microgel has a smaller intensity difference between the different compartments than the aqueous streams in the microfluidic device. This demonstrates that a degree of mixing does occur during gelation, reducing the difference between the compartments.

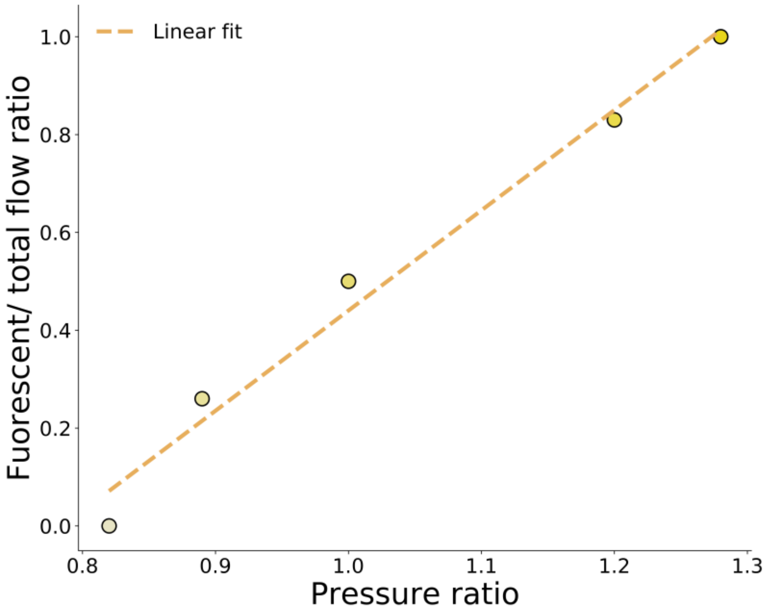

Figure S5: Varying the sizes of the fluorescent/ non-fluorescent aqueous flows through altering the pressure ratio. A graph showing how the pressure ratio impacts the fluorescent/ total flow rate ratio for 1 wt% alginate solutions. On increasing the pressure supplied to the fluorescent aqueous stream, the size of the fluorescent aqueous stream increases while the non-fluorescent aqueous stream size decreases. This increases the size of the fluorescent compartment in the produced compartmentalised microgels. The linear fit show by the dotted line demonstrates the correlation between the altering the pressure and the size of the fluorescent aqueous stream (R^2 value=0.98).

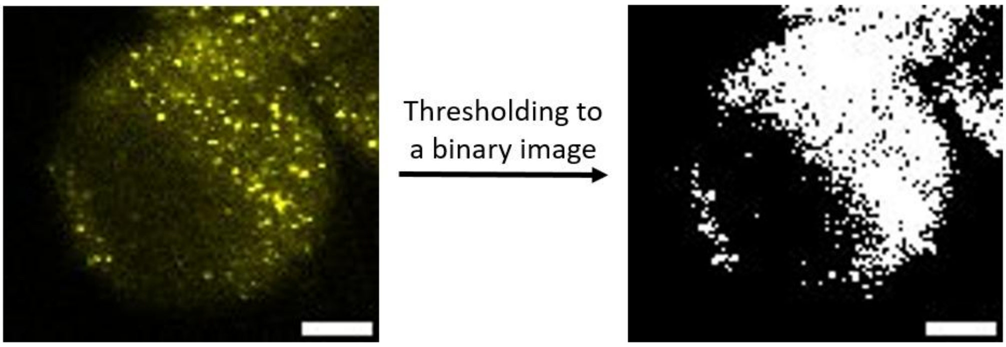

Figure S6: Image thresholding to extract compartment sizes. A fluorescence confocal image of a multicompartment hydrogel was thresholded and converted to a binary image. The fluorescent compartment appeared as the white signal while the non-fluorescent compartment was black. The hydrogel region of interest on the binary image was then measured and the mean signal was used to calculate the fluorescent compartment size. A value of 1 corresponds to a fully fluorescent hydrogel and a value of 0 corresponds to an entirely non-fluorescent hydrogel. This example has a compartment size of 0.48. All hydrogel images were subjected to the same thresholding procedure to compare between populations. The scale bars are 20 µm.

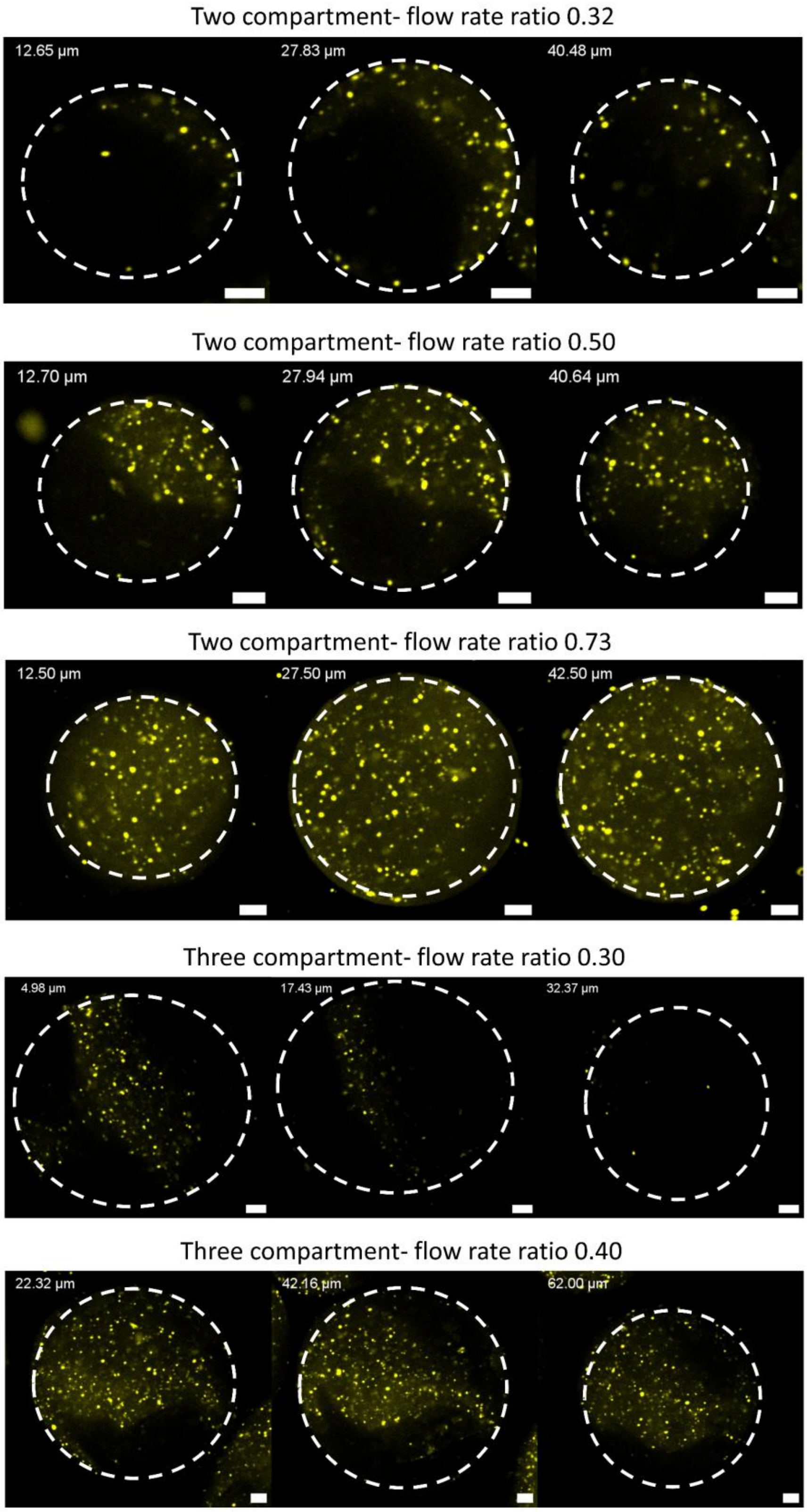

Figure S7: Z stacks of multicompartment hydrogels. Images from Z stacks of two and three compartment hydrogels produced using different flow rate ratios. The fluorescent compartment sizes can be seen to vary slightly at different heights. The dotted line shows the position of the hydrogel in each image. The scale bars are all 10 µm.

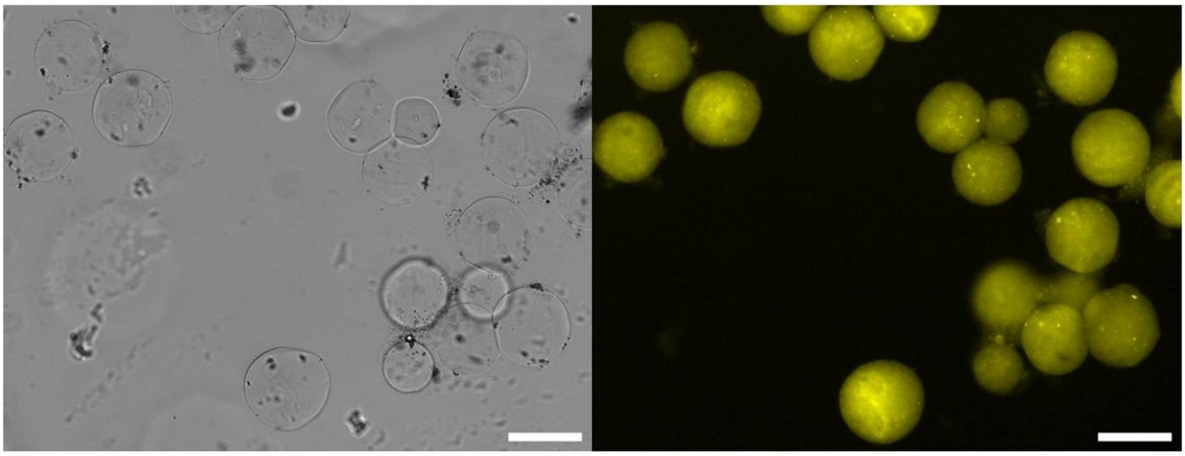

Figure S8: Stability of microgels over extended time periods. Brightfield and fluorescence microscopy images of a population of microgels after 5 months stored in buffer (0.5 M sucrose, 100 mM HEPES (pH 7.4), 100 mM KCl, 20 mM $CaCl_2$) in a fridge. The microgels were comprised of one 4 wt% fluorescent compartment. This shows that the microgels are stable for prolonged periods. The scale bars are 100 µm.

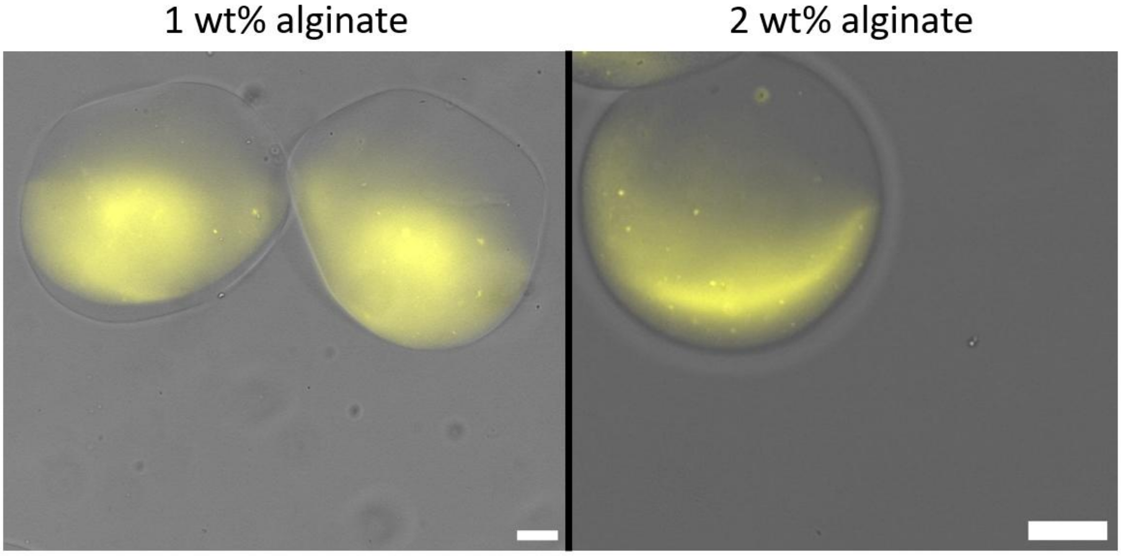

Figure S9: Forming two-compartment microgels with different alginate percentages. Compartmentalised microgels can be formed with both 1 and 2 wt% alginate solutions. The microscopy images are generated by overlaying a fluorescence image of labelled alginate present in one compartment onto a brightfield image of the hydrogel. The scale bars are 20 µm.

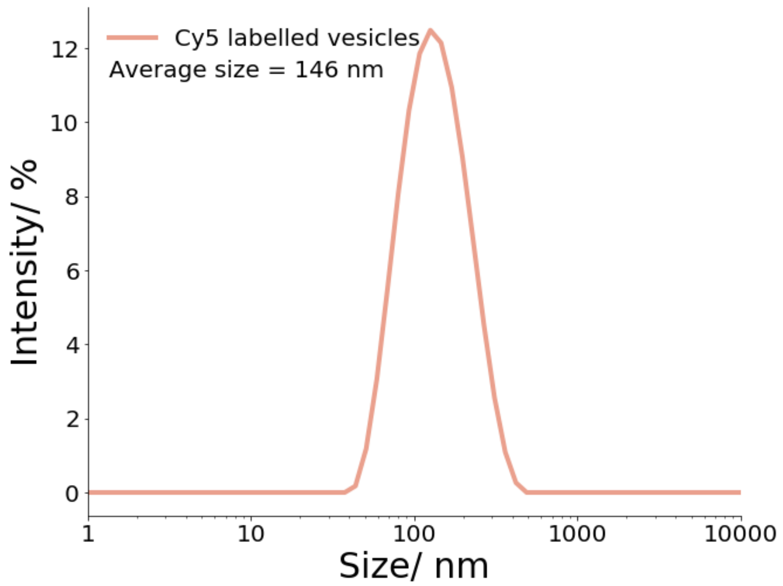

Figure S10: DLS intensity size distribution of a representative extruded vesicle population before insertion into the compartmentalised microgels. The average size of the extruded vesicle population is displayed on the graph. To record the size distribution, the vesicles were diluted in a 1:10 ratio in a buffer containing 100 mM HEPES, 100 mM KCl, 0.5 M Sucrose and 20 mM $CaCl_2$.

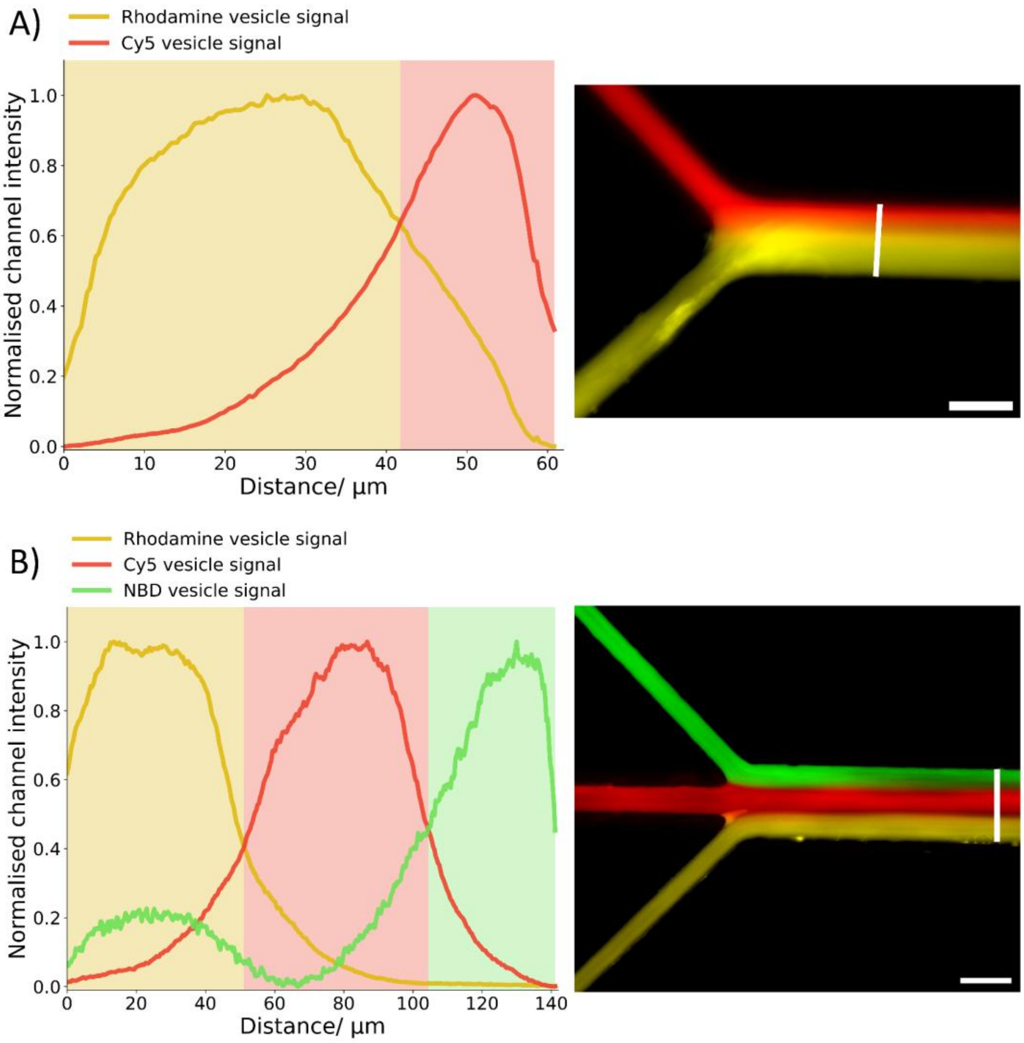

Figure S11: Line profile across vesicle containing streams in a microfluidic device. A) The two different fluorescent signals from the vesicle organelles are prominent in different regions of a two-channel microfluidic device. However, a gradual decrease of both the signals across regions of the line profile is observed, showing some mixing between the streams. The background colour illustrates the prominent vesicle organelle containing stream across sections of the line profile. The line profile was extracted from the white line on the accompanying microscopy image which is comprised of fluorescent signals from the two vesicle organelles containing aqueous streams. The scale bar is 50 µm. **B)** The three different fluorescent signals from the vesicle organelles are prominent in different regions of a three-channel microfluidic device. However, a gradual decrease of the signals across regions of the line profile is observed, showing some mixing between the streams. The interface regions between the different fluorescence maxima were ~ 30 µm. The background colour illustrates the prominent vesicle organelle containing stream across sections of the line profile. The small increase in fluorescence of the NBD signal within the rhodamine region is attributed to spectral overlap between the NBD and rhodamine signals. The line profile was extracted from the white line on the accompanying microscopy image which is comprised of fluorescent signals from the three vesicle organelles containing aqueous streams. The scale bar is 100 µm.

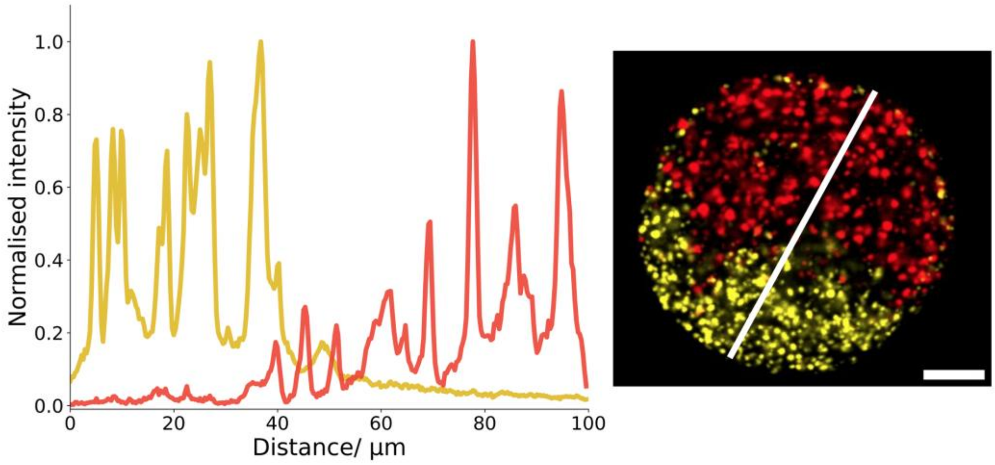

Figure S12: Line profile across a two compartment microgel with the compartments containing fluorescent vesicle organelles. The two different fluorescent signals from the vesicle organelles are prominent in different regions of the microgel, showing that the vesicle organelles can be separated in compartmentalised microgels. The line profile was extracted from the white line on the accompanying microscopy image which is comprised of fluorescent signals from the two vesicle organelle populations. The scale bar is 20 µm.

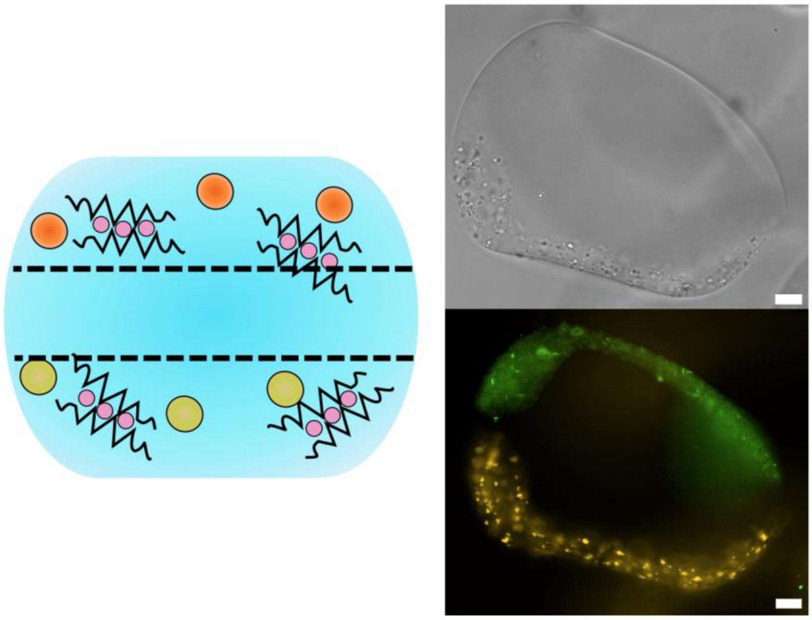

Figure S13: Alternate configurations of three compartment organelle containing microgels. The illustration depicts an elongated three compartment microgel with the compartments containing Rh-PE labelled vesicle organelles (yellow), no vesicle organelles and calcein filled vesicle organelles (orange). The fluorescence and brightfield microscopy images show the successful generation of this compartmentalised microgel architecture. The yellow fluorescent signal is present from the Rh-PE dye and the green fluorescent signal is present from the encapsulated calcein. The scale bar is 20 µm.

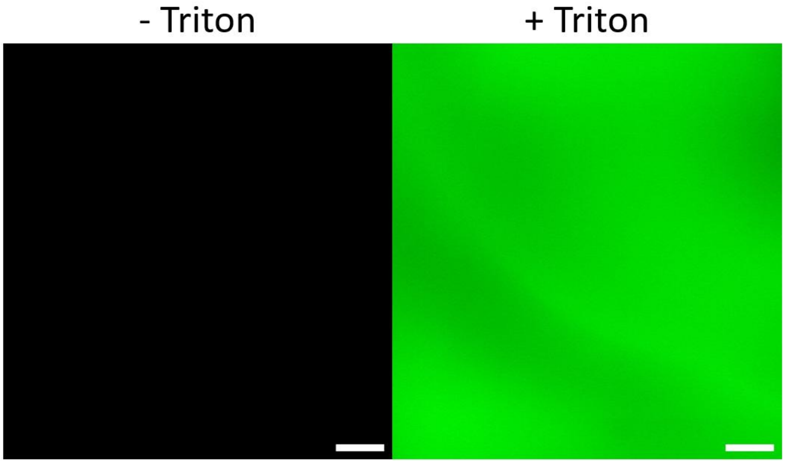

Figure S14: Calcein encapsulation in POPC vesicles. Microscopy images of a population of POPC vesicles containing Calcein before and after the addition of Triton X-100. The addition of Triton X-100 led to vesicle lysis and the release of the trapped quenched Calcein, causing an increase in fluorescent signal and confirming the successful encapsulation of Calcein cargo. The scale bars are 100 µm.

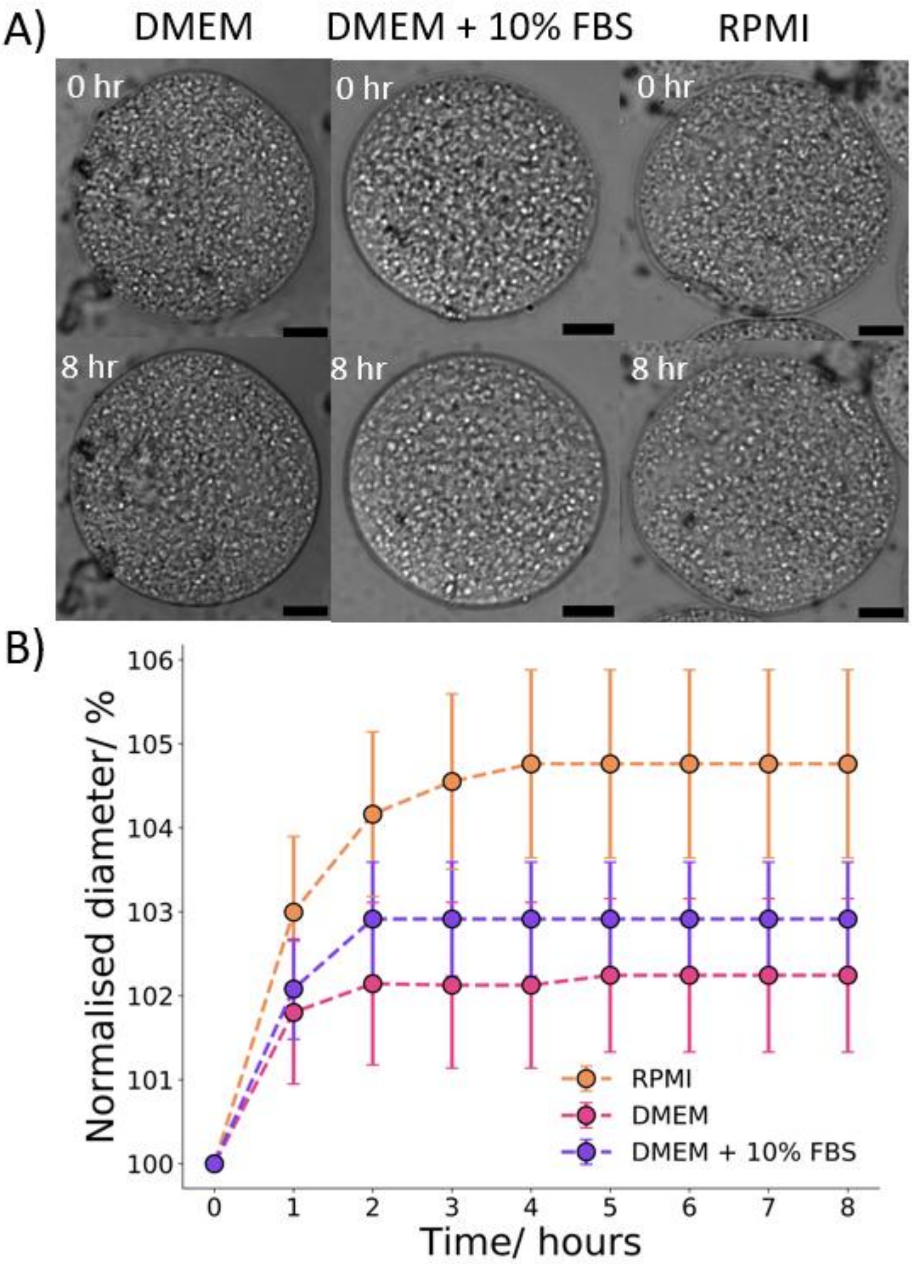

Figure S15: Stability of three compartment organelle containing microgels in different biological buffers. A) Brightfield images of compartmentalised organelle containing microgels in three different biological buffers. Over the 8-hour monitoring period the microgels remained intact and possessed the same optical texture. The scale bars are all 20 µm. **B)** A graph showing the change in size of the organelle containing compartmentalised microgels in different biological buffers. A small change in size was seen due to the different osmolarities of the buffers compared to the storage buffer (0.5 M sucrose, 100 mM HEPES (pH 7.4), 100 mM KCl, 20 mM $CaCl_2$). The error bars are the standard deviation of n=10 microgels.

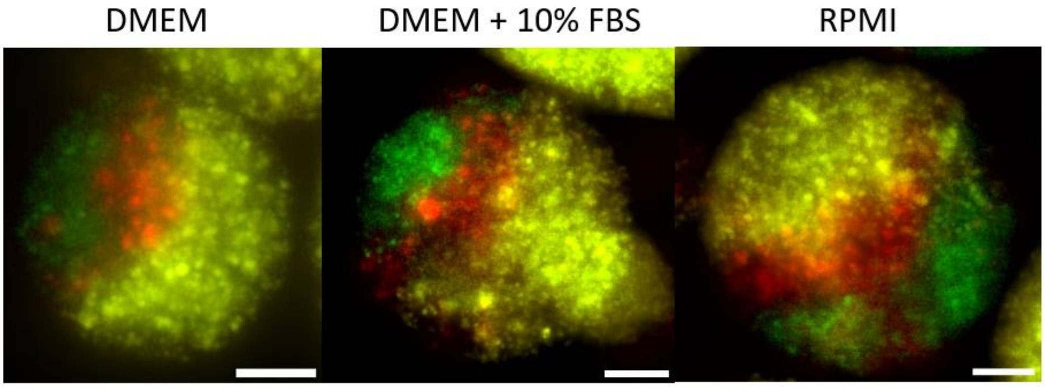

Figure S16: Stability of organelles in three compartment microgels in different biological buffers. Fluorescence microscopy images of three compartment organelle containing microgels after 12 hours in different biological buffers. The images are produced from the overlaying of three different fluorescent signals which show the continued separation of intact organelle populations within the hydrogels, verifying that the microgel organelles are stable in biological buffers. The scale bars are all 20 µm.

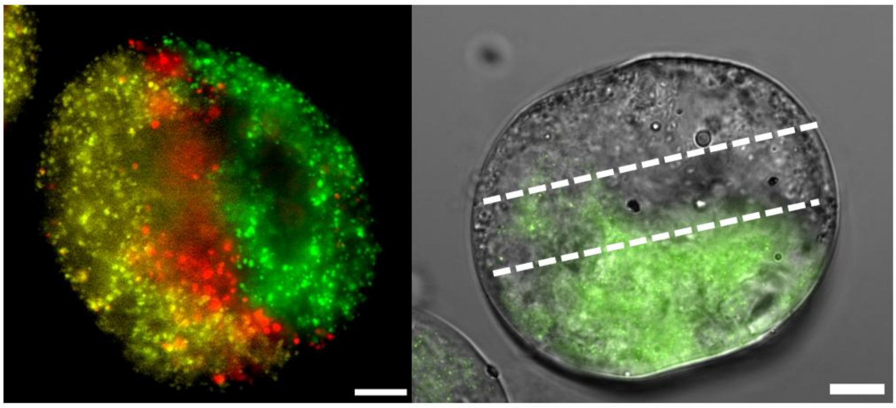

Figure S17: Stability of organelle containing microgels over extended time periods. Images of organelle containing microgels after being stored in buffer (0.5 M sucrose, 100 mM HEPES (pH 7.4), 100 mM KCl, 20 mM $CaCl_2$) in a fridge for 5 months. The lefthand image is produced from the overlaying of three different fluorescent signals in a three-compartment organelle containing microgel. The righthand image is of a three-compartment functional organelle containing microgel and is produced from overlaying the signal from the fluorescent dye remaining trapped in the controlled release vesicles to a brightfield image, the dotted lines show the compartment boundaries. Both images show the organelles in the compartmentalised microgels are stable for prolonged periods. The scale bars are 20 µm.

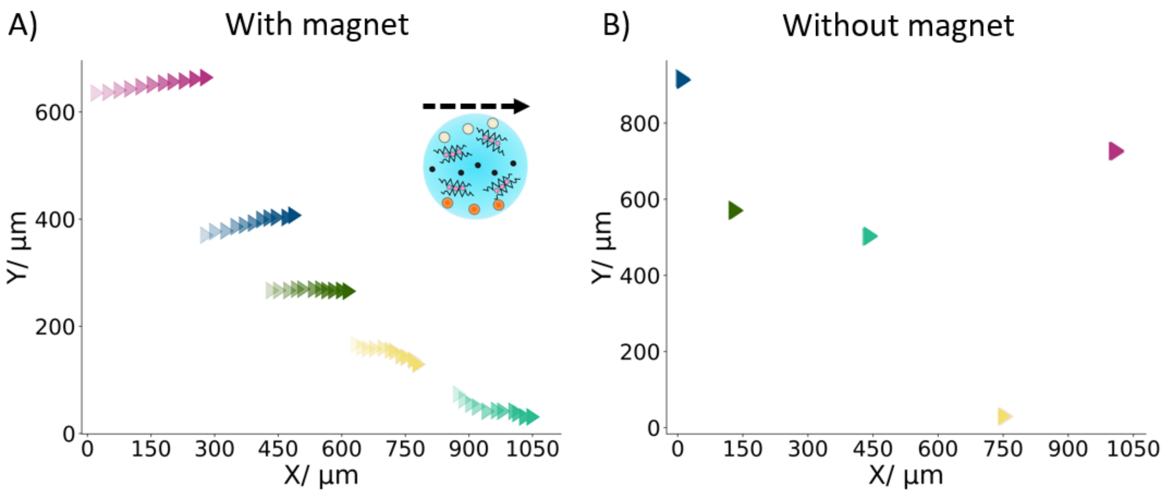

Figure S18: Trajectories of three compartment microgels containing magnetic particle organelles. A) Trajectories of 5 different microgels under the application of a magnet over 10 s. The motion direction is shown by the dashed arrow and the increased colour in the data points. **B)** Trajectories of 5 different microgels without magnet application over 10 s. On magnet application the microgels move in the same direction towards the magnet.

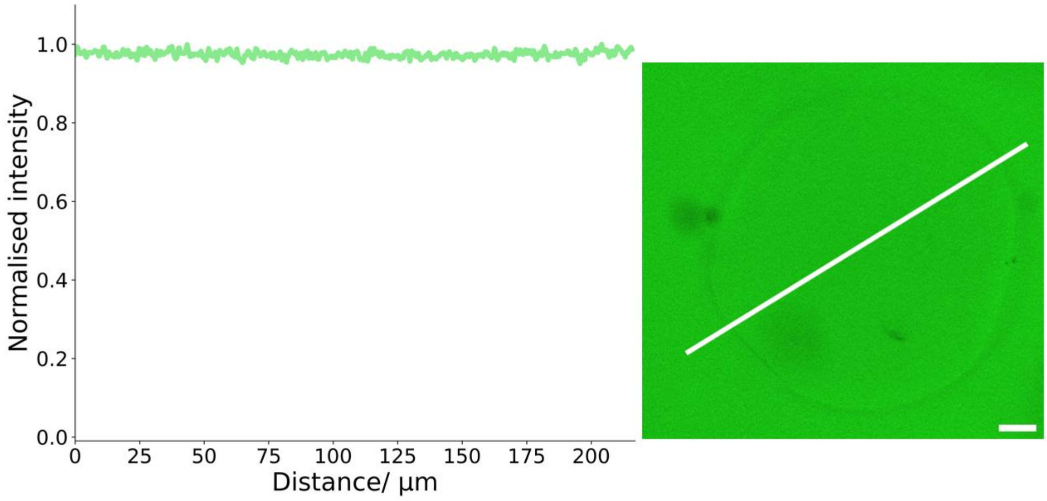

Figure S19: Line profile of Streptavidin permeation throughout a microgel without vesicle organelles. The fluorescent signal from the Streptavidin can be seen to be present throughout the entire microgel at the same intensity. This demonstrates that the Streptavidin freely diffuses into the microgel and does not localise without the presence of Biotin-PE containing vesicle organelles. The line profile was extracted from the white line on the accompanying fluorescence microscopy image. The scale bar is 20 µm.

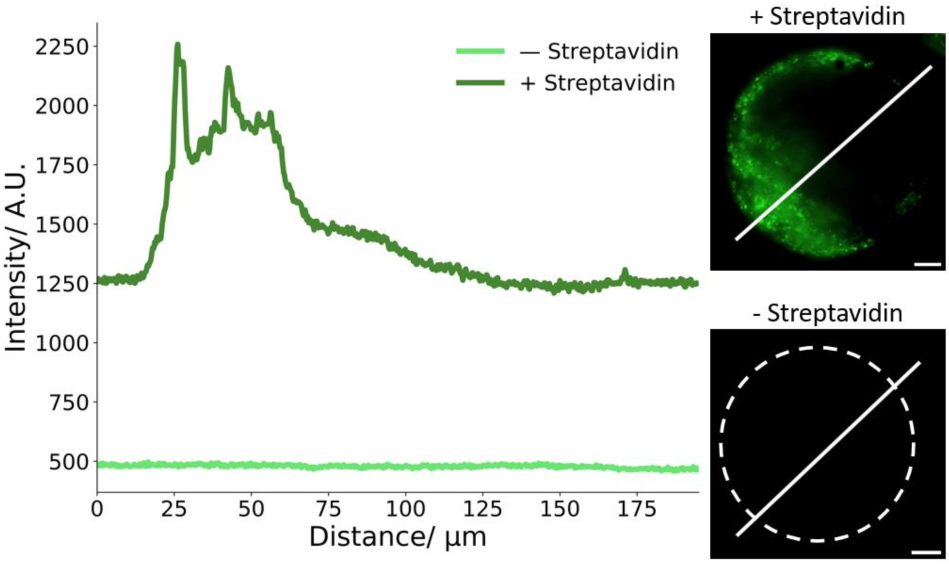

Figure S20: Line profiles comparing fluorescence before and after Streptavidin addition. On addition of Streptavidin the fluorescence of the surrounding environment and the localised signal in the microgels increases significantly. Therefore, confirming that the localised signal can be attributed to Streptavidin binding to the Biotin-PE containing vesicle organelles and not from the vesicle organelles containing self-quenched Calcein. The line profiles were extracted from the white lines on the accompanying fluorescence microscopy images used in Fig. 4F. The dotted circle shows the position of a microgel before Streptavidin addition. The scale bars are 20 µm.

SI video descriptions

Video S1: Brightfield microfluidic manufacture of two compartment microgels.

Video S2: Fluorescence microfluidic manufacture of two compartment microgels.

Video S3: Fluorescence Z stack of a two compartment microgel.

Video S4: Brightfield microfluidic manufacture of three compartment functional microgels.

Video S5: Controlled release of Calcein from vesicle organelles in a three compartment microgel in response to 100 nM $sPLA_2$ addition.

Video S6: Magnetically driven motility of a three compartment microgel.
